# Robust, universal biomarker assay to detect senescent cells in biological specimens

**DOI:** 10.1111/acel.12545

**Published:** 2016-11-17

**Authors:** Konstantinos Evangelou, Nikolaos Lougiakis, Sophia V. Rizou, Athanassios Kotsinas, Dimitris Kletsas, Daniel Muñoz‐Espín, Nikolaos G Kastrinakis, Nicole Pouli, Panagiotis Marakos, Paul Townsend, Manuel Serrano, Jiri Bartek, Vassilis G. Gorgoulis

**Affiliations:** ^1^ Molecular Carcinogenesis GroupDepartment of Histology and EmbryologyMedical SchoolNational and Kapodistrian University of AthensAthensGreece; ^2^ Department of Pharmaceutical ChemistryFaculty of PharmacyNational and Kapodistrian University of AthensAthensGreece; ^3^ Laboratory of Cell Proliferation and AgeingInstitute of Biosciences and ApplicationsNational Centre for Scientific Research ‘Demokritos’AthensGreece; ^4^ Tumor Suppression GroupMolecular Oncology ProgramSpanish National Cancer Research Centre (CNIO)MadridSpain; ^5^ Molecular and Clinical Cancer SciencesManchester Cancer Research CentreManchester Academic Health Sciences CentreUniversity of ManchesterManchesterUK; ^6^ Danish Cancer Society Research CenterCopenhagenDenmark; ^7^ Science For Life LaboratoryDivision of Translational Medicine and Chemical BiologyDepartment of Medical Biochemistry and BiophysicsKarolinska InstituteStockholmSweden; ^8^ Biomedical Research FoundationAcademy of AthensAthensGreece

**Keywords:** aging, biotin‐linked compounds, immunohistochemistry, senescence, Sudan Black B

## Abstract

Cellular senescence contributes to organismal development, aging, and diverse pathologies, yet available assays to detect senescent cells remain unsatisfactory. Here, we designed and synthesized a lipophilic, biotin‐linked Sudan Black B (SBB) analogue suitable for sensitive and specific, antibody‐enhanced detection of lipofuscin‐containing senescent cells in any biological material. This new hybrid histo‐/immunochemical method is easy to perform, reliable, and universally applicable to assess senescence in biomedicine, from cancer research to gerontology.

**Abbreviations**
BCIP/NBT: 5‐Bromo‐4‐Chloro‐3‐Indolyl phosphate/Nitroblue tetrazolium salt
DAB: 3,3′‐Diaminobenzidine
DCC: N,N’‐dicyclohexylcarbodiimide
DMAP: 4‐dimethylaminopyridine
DMF: N,N‐dimethylformamide
HPLC: high‐performance liquid chromatography
NMR: nuclear magnetic resonance
SA‐β‐gal: Senescence‐associated β‐galactosidase
SBB‐A‐B: SBB‐Analogue (GL13) Biotin
SBB: Sudan Black B

Cellular senescence is a fundamental biological process involved in normal embryonic and adult life and implicated in various pathological conditions and therapeutic interventions (Campisi & d'Adda di Fagagna, 2007; Halazonetis *et al*., 2008; Gorgoulis & Halazonetis, 2010; Rodier & Campisi, 2011; Muñoz‐Espín & Serrano, 2014; Georgakopoulou *et al*., 2016). Therefore, detection and measurement of senescent cells in biological material, with a sensitive, precise, and easy‐to‐perform assay would be highly desirable and provide major benefits for research and clinical practice.

We recently reported specific recognition of senescent cells in biological material including cultured cells, fresh/frozen, and archival (formalin‐fixed and paraffin‐embedded, FFPE) tissues, applying the Sudan Black B (SBB) histochemical dye (Georgakopoulou *et al*., 2013; Galanos *et al*., 2016; Liakou *et al*., 2016; Petrakis *et al*., 2016). SBB reacts with lipofuscin, a non degradable aggregate of oxidized proteins, lipids, and metals (Jung *et al*., 2007). Lipofuscin accumulates in senescent cells, as a by‐product of the senescent process, and should be considered as a new ‘hallmark’ of senescence (Jung *et al*., 2007; Georgakopoulou *et al*., 2013; Galanos *et al*., 2016; Liakou *et al*., 2016; Petrakis *et al*., 2016). On the contrary, the most widely used method, measuring senescence‐associated β‐galactosidase activity (SA‐β‐gal; Dimri *et al*., 1995), is applicable only in fresh samples. Additionally, SA‐β‐gal assay has been shown to produce false‐positive results, under certain cell culture conditions (confluence and serum starvation), as well as negative ones in certain cells that fully undergo senescence, but do not exhibit SA‐β‐gal activity (Georgakopoulou *et al*., 2013; Muñoz‐Espín & Serrano, 2014). Hence, the SBB histochemical assay bypasses these weaknesses and broadens the spectrum of applications (Jung *et al*., 2007; Georgakopoulou *et al*., 2013; Muñoz‐Espín & Serrano, 2014; Galanos *et al*., 2016; Liakou *et al*., 2016; Petrakis *et al*., 2016). Although the SBB‐based method is easy and fast, it exhibits some technical challenges that can compromise the overall sensitivity and utilization of the assay, when performed by non pathologists (Georgakopoulou *et al*., 2013).

First, the visualization of SBB‐positive lipofuscin granules that appear as variably sized blue‐black or brown cytoplasmic granules is not always straightforward and requires high magnifications (up to ×1000) for light microscopy. Especially, in FFPE sections, the lipofuscin granules can be very small, due to partial lipid striping of lipofuscin during sample preparation, thereby making the granules hard to detect. If the proportion of senescent cells within a tissue is low and these cells are widely scattered, their identification could be challenging. Second, the SBB staining requires longer experience to become familiar with the detection of positive cells, especially when evaluating the staining reaction under the light microscope without any counter stain. Equally important is to gain experience with discrimination of true SBB‐positive lipofuscin granules in senescent cells from ‘background dirt’, reflecting nonspecific excess and precipitation of the dye that can compromise the analysis.

The preparation of the commercially available SBB dye is based on (E)‐4‐(phenyldiazenyl)naphthalen‐1‐amine (**1**) as the starting material, which is first diazotized and the corresponding diazonium salt (**2**) is then coupled with 2,2‐dimethyl‐2,3‐dihydro‐1H‐perimidine (**3**) (Fig. S1a and Table S1). This procedure results in a complex mixture of compounds, the main derivative of which is 2,2‐dimethyl‐6‐((E)‐(4‐((E)‐(phenyldiazenyl)naphthalen‐1yl)diazenyl)‐2,3‐dihydro‐1H‐perimidine (**4**, approximately 60% of the dye), followed by 2,2‐dimethyl‐4‐((E)‐(4‐((E)‐(phenyldiazenyl)naphthalen‐1yl)diazenyl)‐2,3‐dihydro‐1H‐perimidine (**5**, approximately 20% of the dye; Figs 1a and S1a, Table S1). At the same time, a great number of impurities, consisting of colored and colorless by‐products, have been detected in the SBB dye, independently of its origin (Fig. 1a; Pfüller *et al*., 1977). The complexity of the commercial SBB dye has also been assessed by our group, with the use of HPLC and NMR techniques (Figs 1a and S1b,c). The presence of impurities in the commercial SBB preparations affects not only its staining performance, due to variation in solubility and lipophilicity of each of the components, but also the ability to be conjugated with other substrates.

**Figure 1:**
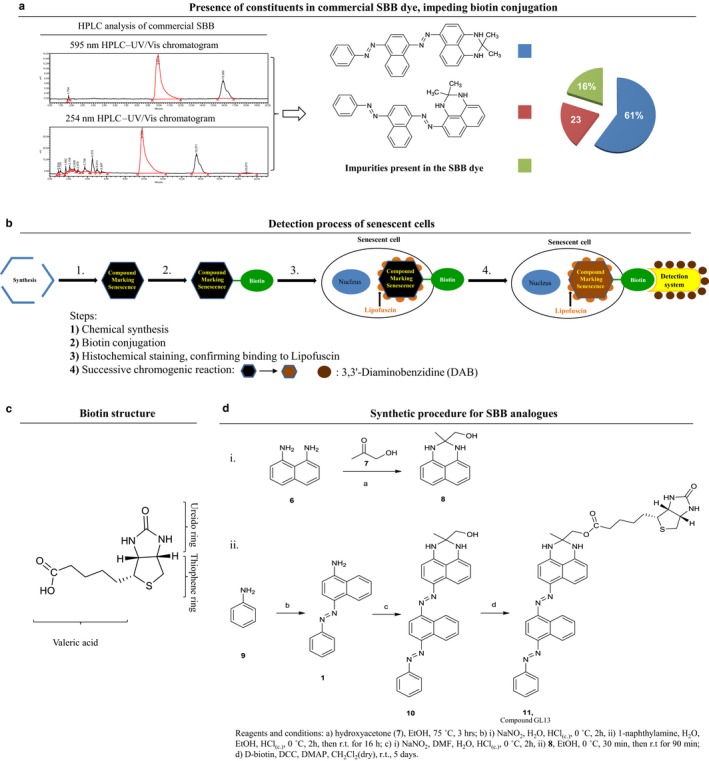
Design and synthesis of a novel chemical compound linked with biotin to detect senescent cells. (a) Nonhomogeneous chemical composition of commercially available SBB dye, as assessed by HPLC analysis (see Fig. S1b,c including NMR data) demonstrating the presence of several constituents that render the SBB dye nonsuitable for chemical modification, affecting also its staining performance. (b) Novel method for senescent cell detection exploiting the specific reaction with lipofuscin of a novel chemical compound linked with biotin. Beyond the histochemical capability of these compounds to stain senescent cells, the presence of biotin allows as a second‐step application of an enhancing immunohistochemical‐enzymatic detection reaction that provides increased sensitivity and recognition precision. (c) Structure of biotin and its particular moieties. (d) Synthesis of compound **11** (**GL13**; see also http://www.gorgoulis.gr/research-activity/main-topics and Appendix S1).

To overcome such technical hurdles, we decided to design and *de novo* synthesize compounds that exhibit structural similarity to SBB (Fig. 1b–d; Appendix S1), but pure, avoiding the disadvantages associated with the numerous by‐products present in the commercial SBB dye (Figs 1a and S1b,c). Next, we chemically coupled our newly synthesized SBB analogues with biotin, a molecule that can be easily visualized by various immunohistochemical, enzymatic‐staining procedures, thus substantially enhancing the applicability and sensitivity of the method (Fig. 1b–d; Appendix S1). The list of chemical structures of our new SBB analogues, and their corresponding esters with biotin, can be found at: http://www.gorgoulis.gr/research-activity/main-topics.

## Experimental design and procedure

The valeric acid side chain of biotin was selected as the reactive center to allow its coupling to the newly synthesized SBB derivatives, so that the ureido and thiophene rings of biotin remain unmodified for binding with streptavidin to take place (Fig. 1c; Diamandis & Christopoulos, 1991; Bolzati *et al*., 2007). The coupling reaction required the existence of a suitable functional group in the molecule of the SBB analogues. To this end, the hydroxyl group was selected as the most appropriate for the development of suitable esters (Fig. 1d, http://www.gorgoulis.gr/research-activity/main-topics). The presence of an ester group did not perturb the lipophilic character of the resulting molecules, thus favoring their affinity for lipofuscin.

SBB itself is highly stable due to its extended aromatic system, and therefore, its targeted chemical modification required *de novo* synthesis of SBB analogues for the link with biotin to be successful (Appendix S1). As a result, three new derivatives with structural similarity to SBB were synthesized, bearing the hydroxyl group either at the 2,3‐dihydro‐1H‐perimidine group of SBB or at the end‐terminal aniline unit (http://www.gorgoulis.gr/research-activity/main-topics, Appendix S1). Subsequently, these alcohols were esterified with D‐biotin providing the corresponding esters (http://www.gorgoulis.gr/research-activity/main-topics, Appendix S1).

The biotinylated compounds were then examined for their ability to react with lipofuscin. They were tested *per se,* as SBB analogues, in various control biological samples, and their staining performance was compared to that of commercially available SSB (at this stage, no enhancing immunohistochemical‐enzymatic detection steps were applied; Figs S2–S4). During this process, compound **11** (termed **GL13** from now on; Fig. 1d; http://www.gorgoulis.gr/research-activity/main-topics) emerged as the most promising derivative, exhibiting comparable, but clearer (with less ‘background dirt’) signal than SBB and was selected for further evaluation.

The synthesis of **GL13** is depicted in Fig. 1d (Appendix S1). Briefly, 1,8‐diaminonaphthalene (**6**) was used as the starting material for the synthesis of the substituted perimidine, which upon treatment with hydroxyacetone (**7**) led to the (2‐methyl‐2,3‐dihydro‐1H‐perimidin‐2‐yl)methanol (**8**) (Fig. 1di, Table S1). Simultaneously, aniline (**9**) was diazotized and then coupled with 1‐naphthylamine, leading to (*E*)‐4‐(phenyldiazenyl)naphthalen‐1‐amine (**1**) (Fig. 1dii, Table S1; Zhang & Zhang, 2014). The latter was diazotized again and coupled with the substituted perimidine **8**, providing the corresponding bis‐diazenyl analogue **10**, which is a hydroxyliated SBB derivative (Table S1, http://www.gorgoulis.gr/research-activity/main-topics). Esterification of this alcohol with D‐biotin, with the use of DCC and DMAP, provided the target compound **11** (**GL13**; Fig. 1dii; Table S1; http://www.gorgoulis.gr/research-activity/main-topics). An analogous D‐biotin linking synthetic procedure was followed for all remaining *de novo‐*synthesized SBB analogues (http://www.gorgoulis.gr/research-activity/main-topics; a detailed description for the synthesis and characterization of all the new compounds is provided in Appendix S1).

**Figure 2:**
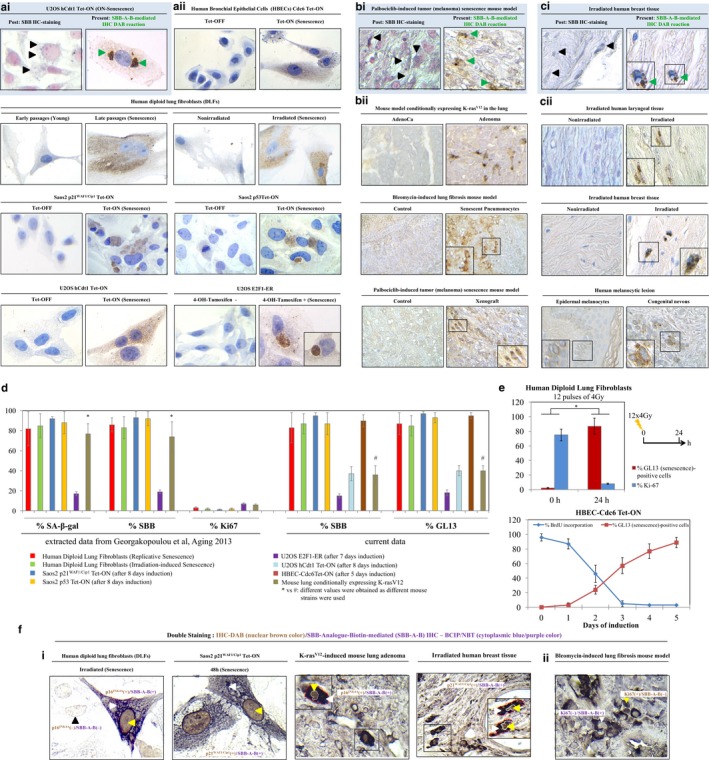
Detection of senescent cells *in vitro* and *in vivo* using a new chemical compound, linked with biotin, and employing an enhancing immunohistochemical‐enzymatic detection assay. Representative results from comparative analysis after applying the SBB histochemical stain (HC) and the hybrid, GL13‐mediated, histo‐/immunochemical assay (HIC), also denoted as SBB‐Analogue‐Biotin (SBB‐A‐B)‐mediated IHC reaction, are shown in panels (ai), (bi), and (ci), (green arrowheads depict GL13 (DAB) staining, while black arrowheads show SBB‐positive granules in the cytoplasm). Specifically, the superiority of the GL‐13 HIC staining relative to SBB HC staining is depicted in the U2OS hCdt1 Tet‐ON cell line (a representative cellular system) (ai) after oncogene (hCdt1)‐induced senescence (OIS), in palbociclib‐induced tumor (human melanoma xenograft) senescence mouse model (a representative animal model) (bi), and in irradiated human breast tissue (a representative human clinical sample) (ci). Further examples of sensitive senescence detection with the GL13 compound, using the HIC assay, in additional cellular systems (aii), mouse models (bii), and human clinical samples (cii) are also shown. Negative controls (devoid of senescent cells) are depicted in each setting. For detailed list of employed models of senescence, see also Fig. S3. (d) Quantitative analysis and concordance of specificity between the SA‐β‐gal, SBB, and GL13 staining, along with inverse relationship with Ki67 positivity in retrospectively (Georgakopoulou *et al*., 2013) and currently examined biological systems with established senescence. (e) Inverse relationship between GL13 HIC staining and proliferation markers (Ki67 and BrdU incorporation) is depicted in human diploid lung fibroblasts (DLFs) and human bronchial epithelial cells (HBEC‐Cdc6 Tet‐ON). (f) Representative images from double‐staining experiments in cellular systems (irradiated DLFs and induced Saos2‐p21^WAF^ ^1/Cip1^), mouse models (K‐ras^V12^‐induced lung adenoma), and human clinical samples (irradiated breast samples) (fi), showing nuclear p16^INK^ ^4A^ or p21^WAF^ ^1/Cip1^ expression (DAB IHC‐brown color: yellow arrowheads) in senescent cells that are concurrently positive with the GL13 compound, visualized with the BCIP/NBT chromogenic hybrid Histo‐IHC reaction (dark blue perinuclear and cytoplasmic color: white arrowheads; red dashed line: cell perimeter; white dashed line: nuclear perimeter; black arrowhead: [P16INK4A(‐)/SBB‐A‐B(‐)]). (fii) Representative image of double staining in lung sections from a mouse model (bleomycin‐induced lung fibrosis) (fii), depicting a strict inverse relationship between nuclear Ki67 positivity and GL13 staining [Ki67(+)/SBB‐A‐B(−): yellow arrowheads; Ki67(−)/SBB‐A‐B(+): white arrowheads]. Magnifications: cells (a,fi) ×630, tissues (b,c) ×400 and (f) ×630; insets ×630. Counterstain (when applied): hematoxylin for HIC and nuclear fast red for HC (SBB).

A two‐step staining procedure was then developed to assess senescence in diverse *in vitro* and *in vivo* settings. The material we investigated included cultured cells, animal models, young versus aged tissues, and clinical scenarios known to trigger or demonstrate robust cellular senescence (Fig. S3, Appendix S2; Bartkova *et al*., 2006; Liontos *et al*., 2007, 2009; Georgakopoulou *et al*., 2013; Galanos *et al*., 2016; Liakou *et al*., 2016; Petrakis *et al*., 2016). In Step 1, we applied the **GL13** compound, *per se,* whereas in Step 2, the antibiotin antibody that carries a peroxidase‐conjugated polymer backbone – 3,3′‐Diaminobenzidine (DAB) to allow enzymatic reaction was included, producing a hybrid histo‐/immunochemical assay (Fig. 1b, Appendix S2). Analyzing the outcome from Step 1, we observed a dark blue to black color of weak‐to‐moderate intensity either in medium‐to‐large‐sized perinuclear structures or in small granules diffusely distributed in the cytoplasm of senescent cells (Fig. S4). Compared to the commercial SBB, the ‘background noise’ was clearly decreased (Fig. S4). Strikingly, the results obtained after completion of Step 2, with the addition of the antibiotin enzymatic detection system, were dramatically improved in both the *in vitro* and the *in vivo* settings (Fig. 2a–c). The reaction from the hybrid histo‐/immunochemical assay (Step 2) produced a clear brown insoluble product reminiscent of standard antibody‐based immunohistochemical reactions (Fig. 2a–c). Negative control cellular systems and tissues (normal or neoplastic), devoid of senescent cells, were completely negative in either the one‐step or two‐step GL13‐based staining procedure, verifying the assay specificity (Fig. 2a–c). Quantifying data from our previous publication and extrapolating it with the results from the current analysis, we found a very strong concordance between SA‐β‐gal, SBB, and GL13 staining in a series of biological materials with strict presence of senescence (Fig. 2d; Georgakopoulou *et al*., 2013). Also, a clear inverse relationship with proliferation markers was established (Figs 2e,f and S5a). Moreover, a precise co‐localization of GL13 with other markers associated with senescence was found employing immunohistochemistry co‐staining (Fig. 2f). GL13 offers senescent cells’ detection versatility as it is also compatible with flow cytometry and immunofluorescence analysis (Fig. S5b,c). Finally, GL13, as SBB staining, was deprived of the false‐positive staining disadvantages of SA‐β‐gal due to serum starvation and cell confluency (Fig. S5d,e; Georgakopoulou *et al*., 2013).

In conclusion, we present an innovative method to detect senescent cells in a wide range of biological materials (cells, fresh/frozen, and importantly archival), based on the design, synthesis, and exploitation of a novel, hapten‐linked, SBB‐inspired analogue. This new compound is a versatile tool to detect senescent cells, with the same specificity as SSB, but with dramatically improved sensitivity and enhanced signal‐to‐noise ratio. Therefore, this novel methodology reported here provides unprecedented advantages over the currently used assays, as it is straight‐forward, sensitive, specific, and widely applicable, even by nonexperienced users. Furthermore, our method can be combined with conventional immunohistochemistry allowing for simultaneous visualization of senescent cells and other, antibody‐defined biomarkers of senescence or other processes. Last but not least, as senescent cells accumulate with age and their genetic or pharmacological (by the so‐called senolytic drugs) elimination was recently shown to rejuvenate tissues and extend health span in animal models (Chang *et al*., 2016), our new method presented here should help monitor the extent of age‐related senescence and the impact of the emerging rejuvenation therapies.

## Funding

Swedish Research Council (Grant/Award Number) Danish National Research Foundation (Grant/Award Number: ‘DNRF125’) Aristeia II GSRT‐Greece (Grant/Award Number: ‘3020’) Inspire FP7‐Capacities (Grant/Award Number: ‘284460’) MRC Confidence in Concept funding UoM R118243 and MRC Proximity to Discovery UoM P2D 010.

## Conflict of interest

*Patents pending*: UK Patent Application Numbers 1611206.2 and 1611208.8, regarding SBB analogues’ chemical synthesis, method(s), and application(s) use.

## Author contributions

NL, NGK, NP, PM, PT and VGG designed and performed chemical synthesis; NL, NP, PM, and PT performed HPLC and NMR analyses; KE, SVR, AK, and NK performed staining reactions in biological material and preparation of biological materials; DK, DME, and MS prepared cell and animal models; KE, NL, PM, JB, and VG conceived and designed the experiments; KE, NL, AK, JB, and VGG prepared figures and manuscript. All authors discussed and interpreted the results.

## Supporting information


**Fig. S1** (a) Chemical synthesis of commercially available SBB dye. (b) HPLC‐UV/Vis chromatograms of the commercial SBB dye, at two different wavelengths, 595 nm and 254 nm. (c) ^1^H NMR spectra of the commercial SBB dye, in three different deuterated solvents.

**Fig. S2** Histo‐/immuno‐chemical (HIC) staining with the SBB analogue GL13 in non‐aged, aged and control tissues.

**Fig. S3** Detailed list of (a) *in vitro* (replicative, stress‐ and oncogene‐induced senescence) and (b) *in vivo* (clinical samples, mice and reference tissues) models of senescence employed to depict the efficiency of the novel compound, described in the present manuscript, to detect senescent cells.

**Fig. S4** Detection of senescent cells *in vitro* and *in vivo,* using the new chemical compound SBB‐A‐B (GL13) ‘*per se*’ and in comparison with the SBB staining.

**Fig. S5** The SBB‐A‐B (GL13) compound detects robustly senescent cells applying various methods and is deprived of the false positive staining disadvantages of SA‐β‐gal.

**Table S1** Correspondence between chemical names and numbers provided in the synthesis processes described in the figures of the manuscript.

**Appendix S1** Chemistry, synthetic experimental procedures and ^1^H NMR, ^13^C NMR and HRMS spectra of the target compounds.

**Appendix S2** Biological material, applied staining procedures and notes.
