# Supplementary material for: Robust, universal biomarker assay to detect senescent cells in biological specimens
Source: Aging Cell. 2016 Nov 17;16(1):192–7. doi: 10.1111/acel.12545 (PMC5242262; doi:10.1111/acel.12545)

Suppl Fig 1

a.

Chemical synthesis of commercial SBB dye

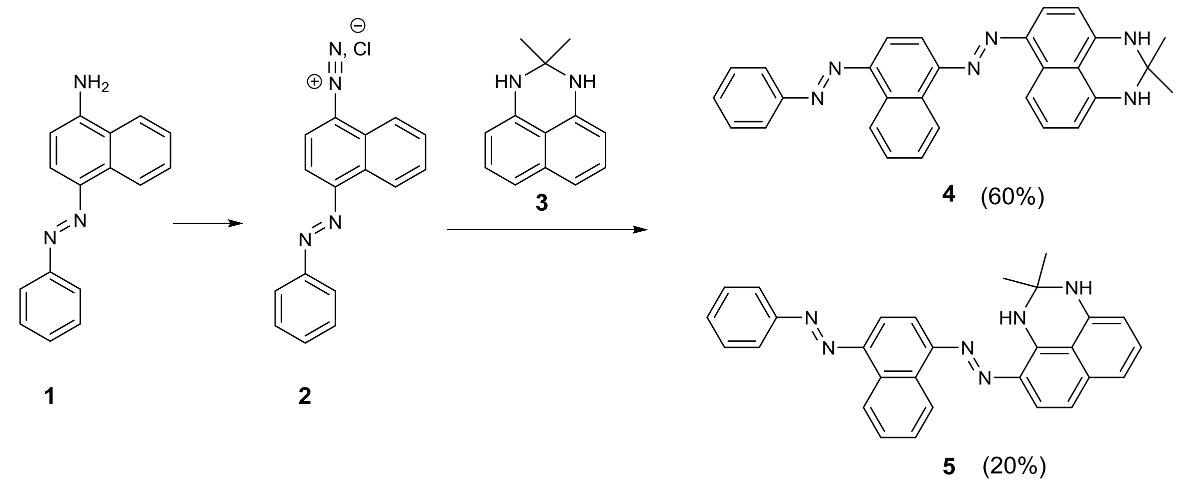

b.

Non-homogeneous chemical composition of commercial SBB dye as revealed by HPLC

i.

595 nm HPLC-UV/Vis chromatogram

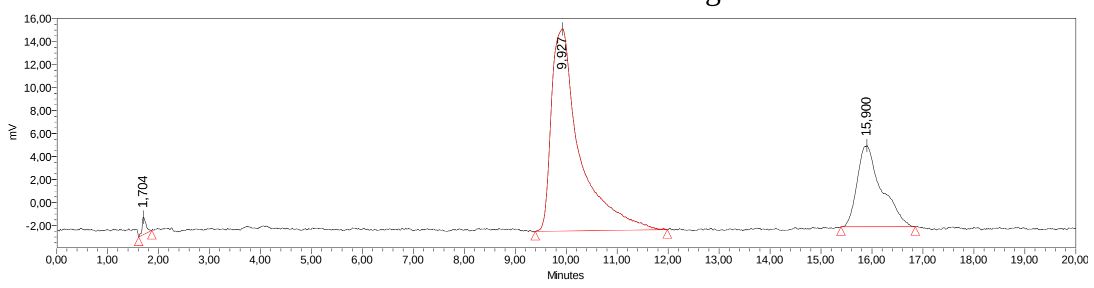

peak	Retention Time	Area	% Area
1	1,704	8116	0,89
2	9,927	675963	73,72
3	15,900	232853	25,39

ii.

254nm HPLC-UV/Vis chromatogram

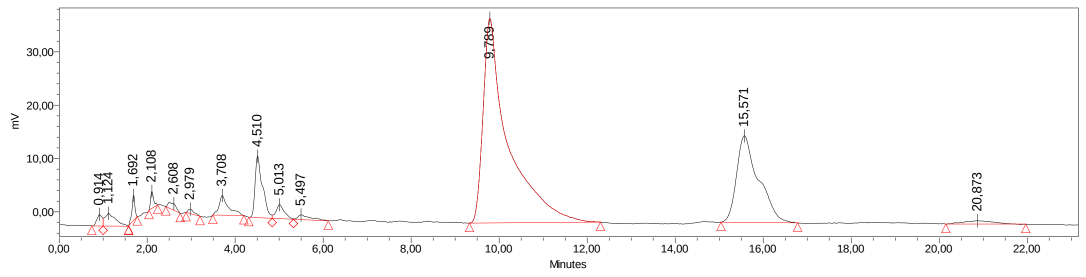

peak	Retention Time	Area	% Area
1	0,914	17583	0,71
2	1,124	40700	1,65
3	1,692	21797	0,89
4	2,108	15302	0,62
5	2,608	14686	0,60
6	2,979	7767	0,32
7	3,708	54800	2,23
8	4,510	134893	5,48
9	5,013	34642	1,41
10	5,497	18429	0,75
11	9,789	1509518	61,32
12	15,571	561857	22,82
13	20,873	29783	1,21

c.

Non-homogeneous chemical composition of commercial SBB dye as revealed by NMR

i.

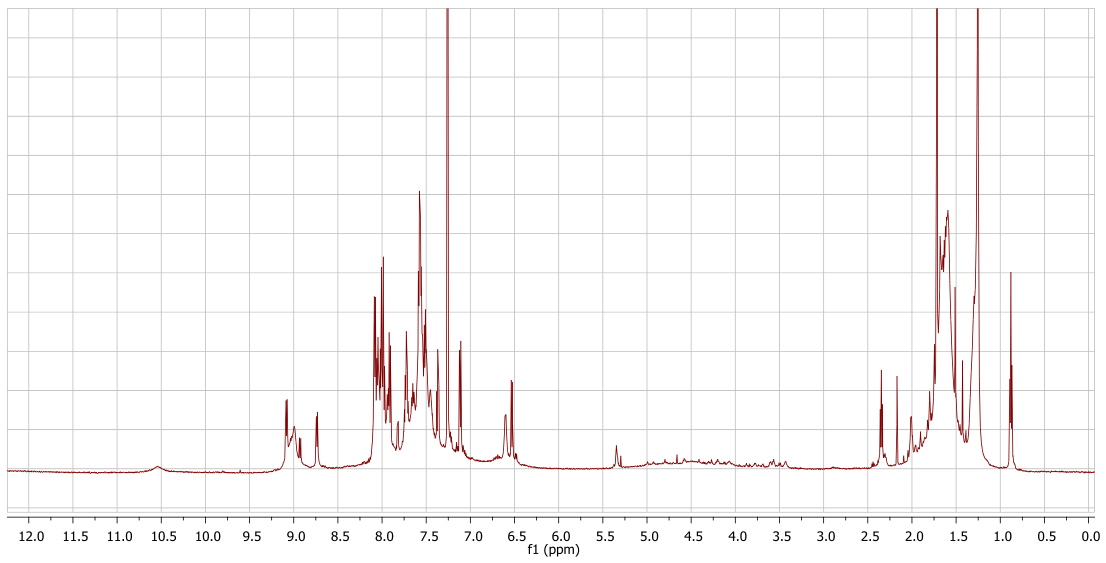

iii.

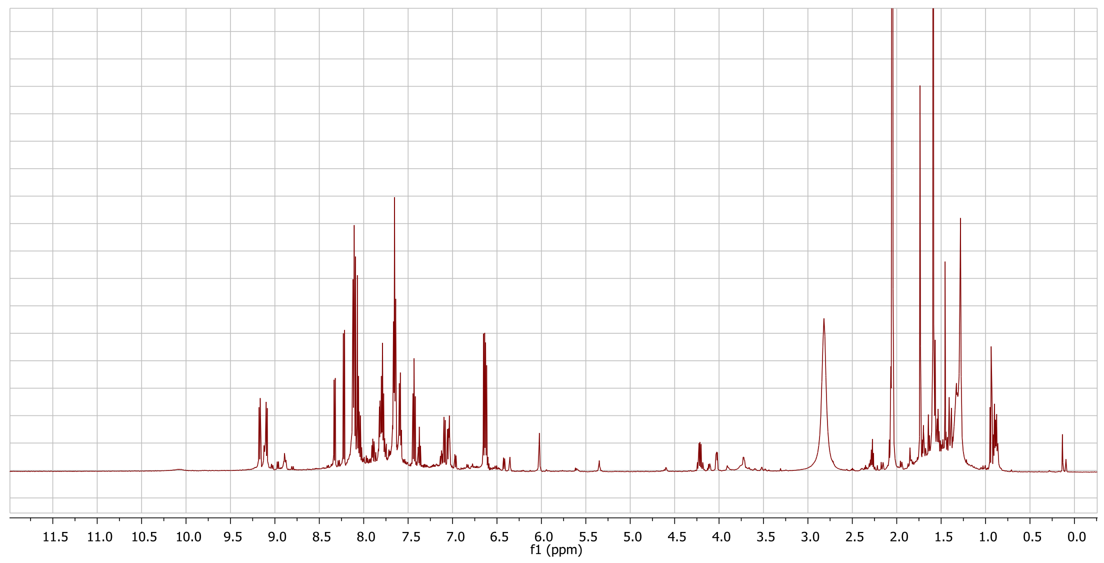

ii.

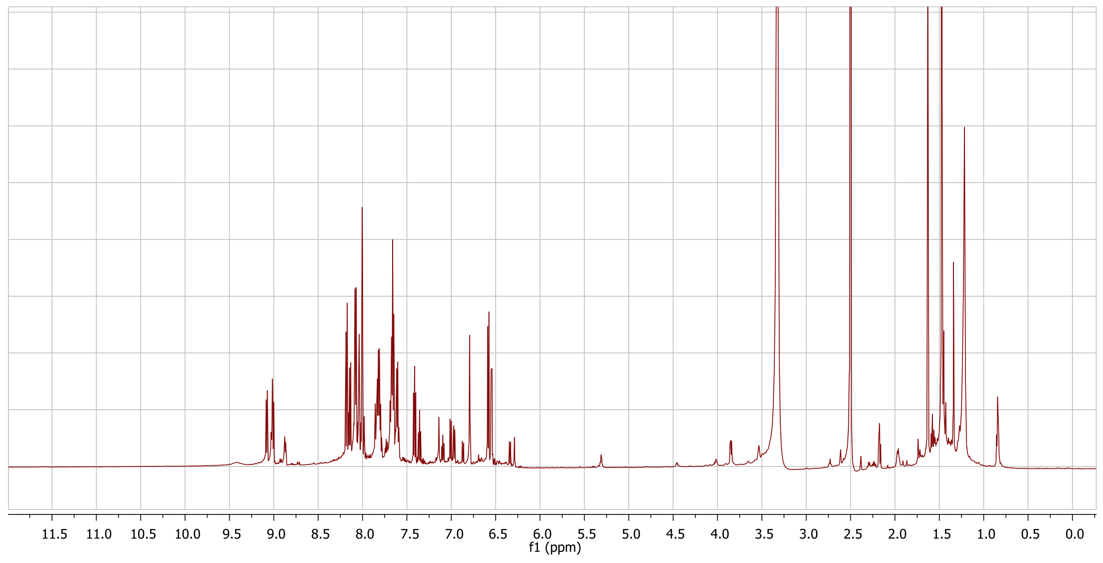

iv.

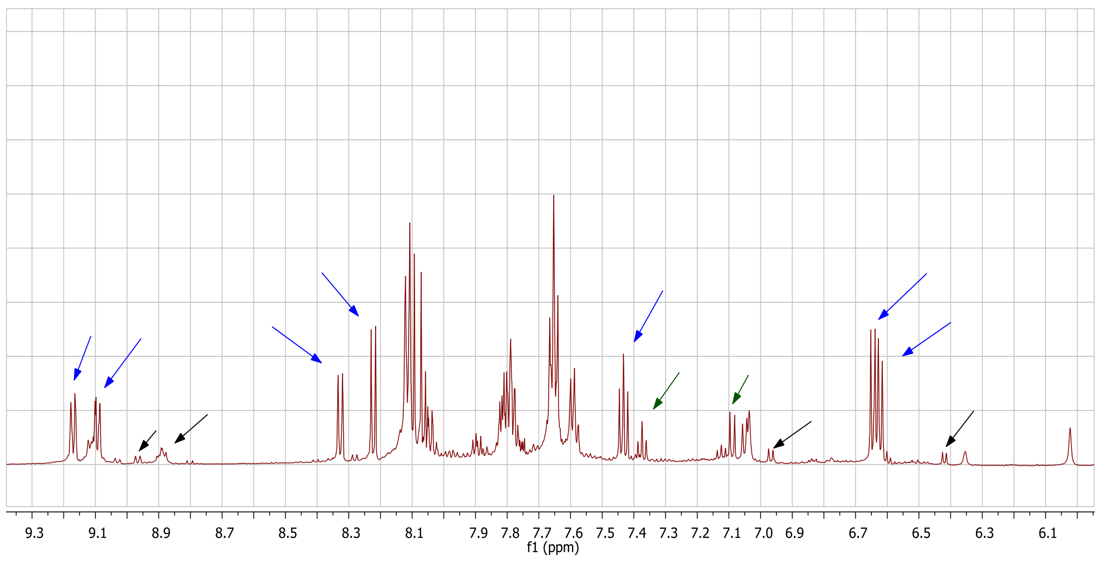

v.

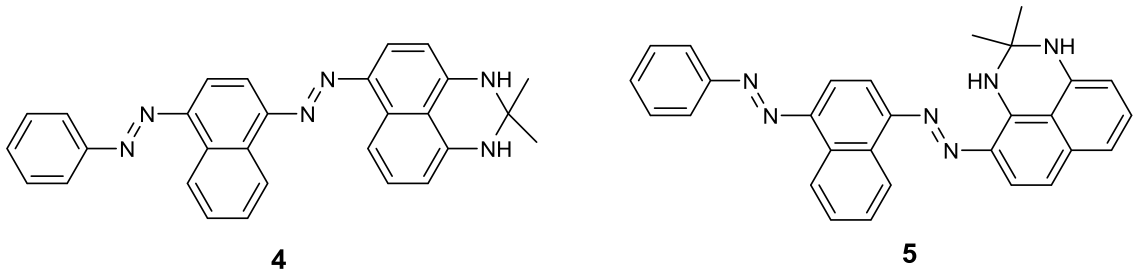

Supplement: Supplementary file 1 — Fig. S1 (a) Chemical synthesis of commercially available SBB dye. (b) HPLC‐UV/Vis chromatograms of the commercial SBB dye, at two different wavelengths, 595 nm and 254 nm. (c) 1H NMR spectra of the commercial SBB dye, in three different deuterated solvents. [file ACEL-16-192-s001.pdf]
