# Supplementary material for: Robust, universal biomarker assay to detect senescent cells in biological specimens
Source: Aging Cell. 2016 Nov 17;16(1):192–7. doi: 10.1111/acel.12545 (PMC5242262; doi:10.1111/acel.12545)
Supplement: Supplementary file 2 — Fig. S2 Histo‐/immuno‐chemical (HIC) staining with the SBB analogue GL13 in non‐aged, aged and control tissues. [file ACEL-16-192-s002.pdf]

Suppl Fig 2

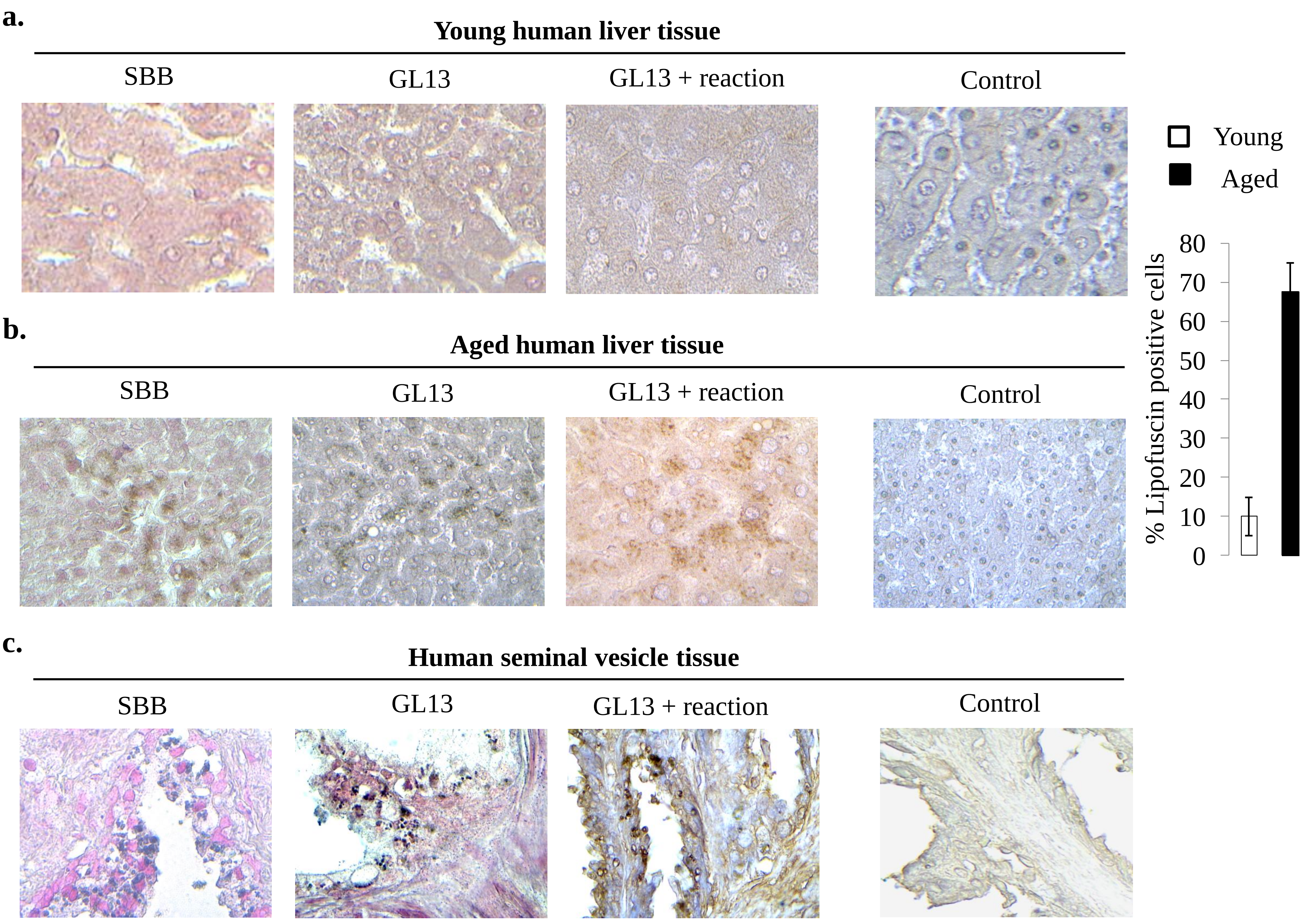
a.
Young human liver tissue
SBB
GL13
GL13 + reaction
Control
b.
Aged human liver tissue
SBB
GL13
GL13 + reaction
Control
c.
Human seminal vesicle tissue
SBB
GL13
GL13 + reaction
Control
Young
Aged
% Lipofuscin positive cells
0
10
20
30
40
50
60
70
80
