# Supplementary material for: Robust, universal biomarker assay to detect senescent cells in biological specimens
Source: Aging Cell. 2016 Nov 17;16(1):192–7. doi: 10.1111/acel.12545 (PMC5242262; doi:10.1111/acel.12545)
Supplement: Supplementary file 3 — Fig. S3 Detailed list of (a) in vitro (replicative, stress‐ and oncogene‐induced senescence) and (b) in vivo (clinical samples, mice and reference tissues) models of senescence employed to depict the efficiency of the novel compound, described in the present manuscript, to detect senescent cells. [file ACEL-16-192-s003.pdf]

Suppl Fig 3

a.

In vitro models

Replicative senescence (RS)

1) Normal human lung diploid fibroblasts
(*early passage vs late passage*)
[Georgakopoulou et al 2013]

Stress Induced Premature Senescence (SISP)

2) Normal human lung diploid fibroblasts
(*irradiated vs non irradiated)*
[Georgakopoulou et al 2013]
3) Saos2-p53 Tet-ON
(*OFF and ON*)
[Georgakopoulou et al 2013]
4) Saos2-p21$^{WAF1/Cip1}$ Tet-ON
(*OFF and ON*)
[Georgakopoulou et al 2013]
[Galanos et al, 2016]

Oncogene Induced Senescence

5) U2OS hCdt1 Tet-ON
(*OFF and ON*)
[Lionto set al 2007]
6) U2OS E2F1-ER
(*OFF and ON*)
[Liontos et al 2009]
7) HBECs-Cdc6 Tet-ON
(*OFF and ON*)
[Petrakis et al 2016]

b.

In vivo models

Clinical samples

1) Head and Neck tumor tissues
(*irradiated vs non irradiated)*
[Evangelou et al 2013]
2) Breast tissues
(*irradiated vs non irradiated)*
[Liakou et al 2013]
3) Congenital nevi vs normal skin
[Michaloglou et al 2005]

Animal models

1) Mouse model expressing
conditionally *K-rasV12*
[Collado et al 2005]
2) Mouse model of bleomycin
induced pneumopathy
[Le et al 2010]
3) Palbociclib treated mouse tumor
melanoma xenograft
[Yoshida et al 2016]

Non-aged, Aged and control tissues

1) Human liver tissue
from young and aged patients
2) Human seminal vesicles
[Evangelou et al 2013]
