# Supplementary figures and images for: Robust, universal biomarker assay to detect senescent cells in biological specimens
Source: Aging Cell. 2016 Nov 17;16(1):192–7. doi: 10.1111/acel.12545 (PMC5242262; doi:10.1111/acel.12545)

Suppl Fig 4

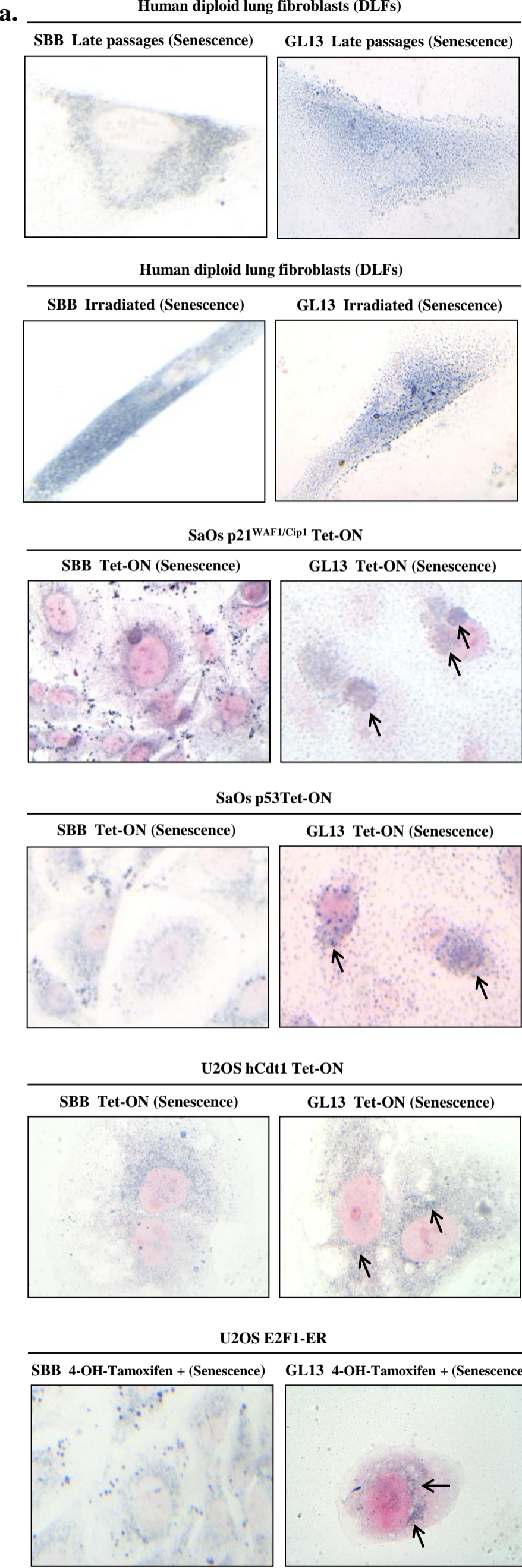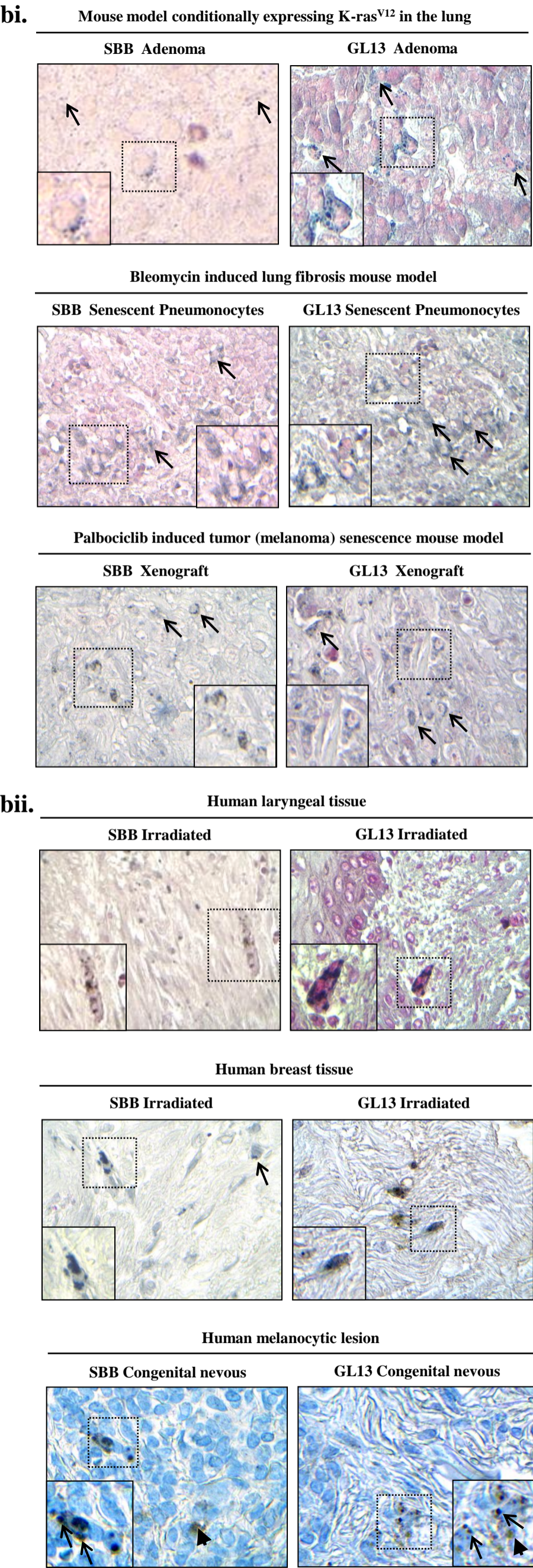

Supplement: Supplementary file 4 — Fig. S4 Detection of senescent cells in vitro and in vivo, using the new chemical compound SBB‐A‐B (GL13) ‘per se’ and in comparison with the SBB staining. [file ACEL-16-192-s004.pdf]
