# Supplementary material for: Robust, universal biomarker assay to detect senescent cells in biological specimens
Source: Aging Cell. 2016 Nov 17;16(1):192–7. doi: 10.1111/acel.12545 (PMC5242262; doi:10.1111/acel.12545)
Supplement: Supplementary file 5 — Fig. S5 The SBB‐A‐B (GL13) compound detects robustly senescent cells applying various methods and is deprived of the false positive staining disadvantages of SA‐β‐gal. [file ACEL-16-192-s005.pdf]

Suppl Fig 5

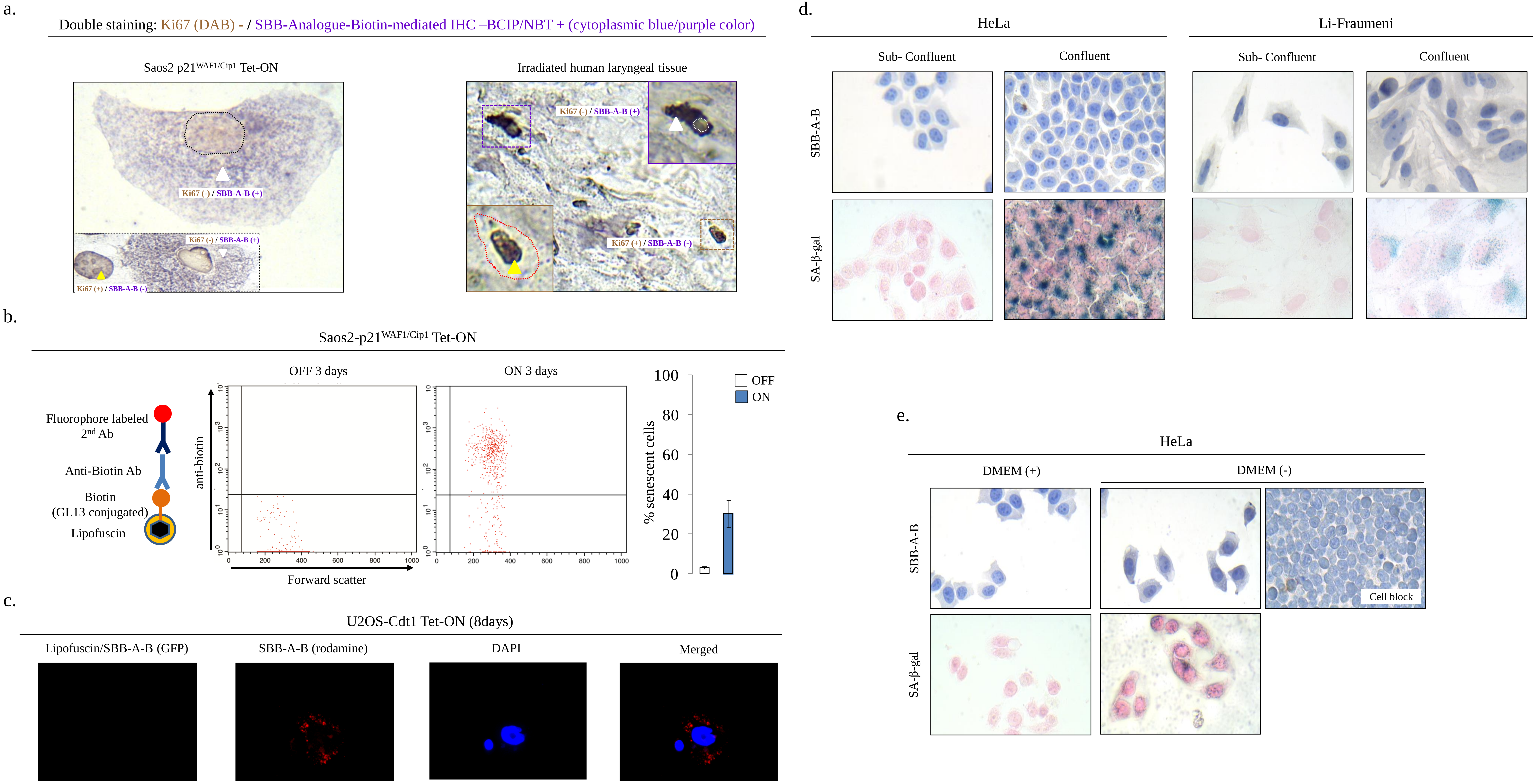

a.
Double staining: Ki67 (DAB) - / SBB-Analogue-Biotin-mediated IHC –BCIP/NBT + (cytoplasmic blue/purple color)
Saos2 p21WAF1/Cip1 Tet-ON
Ki67 (-) / SBB-A-B (+)
Ki67 (-) / SBB-A-B (+)
Ki67 (+) / SBB-A-B (-)
Irradiated human laryngeal tissue
Ki67 (-) / SBB-A-B (+)
Ki67 (+) / SBB-A-B (-)
b.
Saos2-p21WAF1/Cip1 Tet-ON
Fluorophore labeled
2nd Ab
Anti-Biotin Ab
Biotin
(GL13 conjugated)
Lipofuscin
OFF 3 days
ON 3 days
anti-biotin
Forward scatter
0
200
400
600
800
1000
% senescent cells
100
80
60
40
20
0
OFF
ON
c.
U2OS-Cdt1 Tet-ON (8days)
Lipofuscin/SBB-A-B (GFP)
SBB-A-B (rodamine)
DAPI
Merged
d.
HeLa
Li-Fraumeni
Sub- Confluent
Confluent
Sub- Confluent
Confluent
SBB-A-B
SA-β-gal
e.
HeLa
DMEM (+)
DMEM (-)
SBB-A-B
SA-β-gal
Cell block
