# Supplementary material for: Robust, universal biomarker assay to detect senescent cells in biological specimens
Source: Aging Cell. 2016 Nov 17;16(1):192–7. doi: 10.1111/acel.12545 (PMC5242262; doi:10.1111/acel.12545)
Supplement: Supplementary file 6 — Table S1 Correspondence between chemical names and numbers provided in the synthesis processes described in the figures of the manuscript. [file ACEL-16-192-s006.doc]

**Supplementary Table 1.** Correspondence between chemical names and numbers provided in the synthesis processes described in the figures of the manuscript.

| **Chemical No** | **Chemical name** |
| --- | --- |
| (1) | (*E*)-4-(phenyldiazenyl)naphthalen-1-amine |
| (2) | (*E*)-4-(phenyldiazenyl)naphthalene-1-diazonium chloride |
| (3) | 2,2-dimethyl-2,3-dihydro-1*H*-perimidine |
| (4) | 2,2-dimethyl-6-((*E*)-(4-((*E*)-(phenyldiazenyl)naphthalen-1yl)diazenyl)-2,3-dihydro-1*H*-perimidine |
| (5) | 2,2-dimethyl-4-((*E*)-(4-((*E*)-(phenyldiazenyl)naphthalen-1yl)diazenyl)-2,3-dihydro-1*H*-perimidine |
| (6) | 1,8-diaminonaphthalene |
| (7) | hydroxyacetone |
| (8) | (2-methyl-2,3-dihydro-1*H*-perimidin-2-yl)methanol |
| (9) | aniline |
| (10) | (2-methyl-6-((*E*)-(4-((*E*)-phenyldiazenyl)naphthalen-1-yl)diazenyl)-2,3-dihydro-1*H*-perimidin-2-yl)methanol |
| (11) | (2-methyl-6-((*E*)-(4-((*E*)-phenyldiazenyl)naphthalen-1-yl)diazenyl)-2,3-dihydro-1*H*-perimidin-2-yl)methyl 5-((3a*R*,4*R*,6a*S*)-2-oxohexahydro-1*H*-thieno[3,4-*d*]imidazol-4-yl)pentanoate |
