# Supplementary material for: Robust, universal biomarker assay to detect senescent cells in biological specimens
Source: Aging Cell. 2016 Nov 17;16(1):192–7. doi: 10.1111/acel.12545 (PMC5242262; doi:10.1111/acel.12545)
Supplement: Supplementary file 7 — Appendix S1 Chemistry, synthetic experimental procedures and 1H NMR, 13C NMR and HRMS spectra of the target compounds. [file ACEL-16-192-s007.doc]

**Supplementary Information 1**

**Chemistry, synthetic experimental procedures and ^1^H NMR, ^13^C NMR and HRMS spectra of the target compounds.**

**Chemistry.**

1,8-Diaminonaphthalene (**6**) was used as the starting material for the synthesis of the substituted perimidines. Thus, treatment of **6** with the appropriate hydroxyketone (hydroxyacetone, 4-hydroxy-2-butanone or 5-hydroxy-2-pentanone) led to the 2-methyl-2,3-dihydro-1*H*-perimidin-2-yl alcohols **8, 12** and **13**, respectively (scheme 1). Compound **13** has been previously reported, using a slightly modified procedure (Crissali & Kool, 2011), however, the method reported herein provides the derivative in almost quantitative yield. Treatment of **6** with 4-hydroxybenzaldehyde afforded the 4-(2,3-dihydro-1*H*-perimidin-2-yl)phenol (**14**), while 2,2-dimethyl-2,3-dihydro-1*H*-perimidine (**3**) was synthesized upon reaction of **6** with acetone (scheme 1).

**Scheme 1**. Reagents and conditions: a) hydroxyacetone for **8**, or 4-hydroxy-2-butanone for **12**, ethanol, 75 ˚C, 3 hrs, or 5-hydroxy-2-pentanone for **13**, ethanol, reflux, 16 hrs; b) 4-hydroxybenzaldehyde, ethanol, reflux, 40 min; c) acetone, r.t., 4 days.

For the preparation of the target compounds **11**, **17** and **18**, aniline (**9**) was first diazotized and then coupled with 1-naphthylamine, leading to (*E*)-4-(phenyldiazenyl)naphthalen-1-amine (**1**, scheme 2) (Zhang & Zhang, 2014). The latter was diazotized again and coupled with the substituted perimidines **8**, **12** and **13**, providing the corresponding bis-diazenyl analogues **10**, **15** and **16**, respectively. Esterification of these alcohols with D-biotin, with the use of DCC and DMAP, provided the target compounds **11**, **17** and **18**.

**Scheme 2**. Reagents and conditions: a) i) NaNO_2_, H_2_O, HCl_(c.)_, 0 ˚C, 2h, ii) 1-naphthylamine, H_2_O, EtOH, HCl_(c.)_, 0 ˚C, 2h, then r.t. for 16 h; b) i) NaNO_2_, DMF, H_2_O, HCl_(c.)_, 0 ˚C, 2h, ii) **8** for the synthesis of **10**, or **12** for the synthesis of **15**, or **13** for the synthesis of **16**, EtOH, 0 ˚C, 30 min, then r.t for 90 min; c) D-biotin, DCC, DMAP, CH_2_Cl_2_(dry), r.t., 5 days.

Following an analogous synthetic procedure, the target compound **20** was prepared, upon reaction of the intermediate **1** with perimidine **14**, and subsequent esterification of the derived alcohol **19** with D-biotin, in the presence of DCC and DMAP (scheme 3).

**Scheme 3**. Reagents and conditions: a) i) NaNO_2_, DMF, H_2_O, HCl_(c.)_, 0 ˚C, 2h, ii) **14**, EtOH, 0 ˚C, 30 min, then r.t for 90 min; b) D-biotin, DCC, DMAP, CH_2_Cl_2_(dry), r.t., 5 days.

For the synthesis of the target compound **24**, 2-(4-aminophenyl)ethanol (**21**) was used as starting material and upon two subsequent diazotisation reactions, the derived alcohol **23** was finally esterified with D-biotin leading to **24** (scheme 4).

**Scheme 4**. Reagents and conditions: a) i) NaNO_2_, H_2_O, HCl_(c.)_, 0 ˚C, 2h, ii) 1-naphthylamine, H_2_O, EtOH, HCl_(c.)_, 0 ˚C, 2h, then r.t. for 16 h; b) i) NaNO_2_, DMF, H_2_O, HCl_(c.)_, 0 ˚C, 2h, ii) **3**, EtOH, 0 ˚C, 30 min, then r.t for 90 min; c) D-biotin, DCC, DMAP, CH_2_Cl_2_(dry), r.t., 5 days.

**Experimental section.**

**General Information**

Melting points were determined on a Büchi apparatus and are uncorrected. ^1^H NMR spectra, ^13^C NMR spectra and 2D spectra were recorded on a Bruker Avance III 600 instrument, in deuterated solvents and were referenced to TMS (*δ* scale). Mass spectra were recorded with a LTQ Orbitrap Discovery instrument, possessing an Ionmax ionization source. Flash chromatography was performed on Merck silica gel 60 (0.040­­­­–0.063 mm). Analytical thin layer chromatography (TLC) was carried out on precoated (0.25 mm) Merck silica gel F-254 plates. The purity of all the synthesized compounds was >95% as ascertained by elemental analysis. Elemental analyses were undertaken using a PerkinElmer PE 240C elemental analyzer (Norwalk, CT, U.S.) and the measured values for C, H, and N were within ±0.4% of the theoretical values. 1-Naphthylamine and 5-hydroxy-2-pentanone were purchased from Sigma-Aldrich, while the rest of the reagents were purchased from Alfa-Aesar, and all of them were used with no further purification.

**General procedure for the synthesis of perimidines 8, 12** and **13.**

The appropriate hydroxyketone (15 mmol) was added into a solution of 1,8-diaminonaphthalene (**6**, 1.58 g, 10 mmol) in absolute ethanol (10 mL) and the resulting mixture was heated at 75˚ C for 3 h in the case of **8** and **12**, or refluxed for 16 h in the case of **13**. Upon completion of the reaction the solvent was evaporated, water (50 mL) was added and the residue was extracted with dichloromethane (3 x 50 mL) for **8** and **12**, or with ethyl acetate (3 x 50 mL) for **13**. The combined organic layers were washed with a saturated solution of sodium chloride (100 mL), dried over sodium sulfate and the solvent was evaporated to dryness to provide pure perimidines **8**, **12** and **13**, which were used to the next step with no further purification.

**(2-Methyl-2,3-dihydro-1*H*-perimidin-2-yl)methanol (8).**

This compound was synthesized according to the general procedure described above, upon reaction of 1,8-diaminonaphthalene with hydroxyacetone, in 89% yield. Beige solid. Mp 126–7 ^o^C. ^1^H NMR (600 MHz, CDCl_3_) δ 1.46 (s, 3H), 3.62 (s, 2H), 3.60-3.80 (brs, 2H, D_2_O exch.), 6.57 (d, 2H, *J*=7.2 Hz), 7.20 (d, 2H, *J*=7.5 Hz), 7.24 (t, 2H, *J*=8.2 Hz). ^13^C NMR (151 MHz, CDCl_3_) δ 24.69, 67.06, 67.13, 107.10, 113.34, 118.02, 127.18, 134.69, 139.20.

**2-(2-Methyl-2,3-dihydro-1*H*-perimidin-2-yl)ethanol (12).**

This compound was synthesized according to the general procedure described above, upon reaction of 1,8-diaminonaphthalene with 4-hydroxy-2-butanone, in 95% yield. Beige solid. M.p. 132–3 ^o^C. ^1^H NMR (600 MHz, acetone-*d_6_*) δ 1.46 (s, 3H), 2.00 (t, 2H, *J*=6.5 Hz), 3.70 (brs , 1H, D_2_O exch.), 3.85 (m, 2H), 5.65 (brs, 2H, D_2_O exch.), 6.44 (d, 2H, *J*=7.4 Hz), 6.99 (d, 2H, *J*=7.9 Hz), 7.14 (t, 2H, *J*=7.8 Hz). ^13^C NMR (151 MHz, acetone-*d_6_*) δ 26.85, 43.56, 59.06, 67.06, 105.74, 113.53, 116.37, 127.92, 135.76, 142.42.

**3-(2-Methyl-2,3-dihydro-1*H*-perimidin-2-yl)propan-1-ol (13).**

This compound was synthesized according to the general procedure described above, upon reaction of 1,8-diaminonaphthalene with 5-hydroxy-2-pentanone, in 97% yield. Grey solid. M.p. 149–150 ^o^C. ^1^H NMR (600 MHz, acetone-*d_6_*) δ 1.43 (s, 3H), 1.68-1.74 (m, 2H), 1.79-1.83 (m, 2H), 3.45-3.53 (m, 3H), 5.61 (brs, 2H, D_2_O exch.), 6.43 (d, 2H, *J*=7.4 Hz), 6.95 (d, 2H, *J*=8.1 Hz), 7.12 (t, 2H, *J*=7.9 Hz). ^13^C NMR (151 MHz, acetone-*d_6_*) δ 27.20, 28.28, 38.32, 62.90, 67.07, 105.35, 113.48, 116.06, 127.93, 135.82, 142.71.

**General procedure for the synthesis of compounds 10, 15 and 16.**

(*E*)-4-(Phenyldiazenyl)naphthalen-1-amine (**1**, 0.5 g, 2.02 mmol) was dissolved in DMF (2 mL) and then H_2_O (3 mL) and HCl (10N, 0.6 mL) were added. This mixture was cooled at 0 ^o^C and then an aqueous solution (1 mL) of NaNO_2_ (139 mg, 2.02 mmol) was added dropwise over a period of 5 minutes. The diazonium salt was stirred at 0^o^ C for 2 hours and then was added dropwise into a beaker containing perimidines **8**, **12 or 13** (2 mmol) in ethanol (8 mL) under vigorous stirring at 0^o^ C. The reaction mixture was stirred at 0^o^ C for 30 minutes and then at room temperature for 90 minutes. The solution was then neutralized with addition of saturated solution of NaHCO_3_ and the resulting dark precipitate was left at 0^o^ C for 60 minutes and then filtered under vacuum, washed with H_2_O and air dried. The crude product was purified by column chromatography (silica gel) to provide pure alcohols **10**, **15** or **16**.

**(2-Methyl-6-((*E*)-(4-((*E*)-phenyldiazenyl)naphthalen-1-yl)diazenyl)-2,3-dihydro-1*H*-perimidin-2-yl)methanol (10).**

This compound was prepared according to the general procedure described above, upon reaction of **1** with perimidine **8**. The crude product was purified by column chromatography using a mixture of dichloromethane / ethyl acetate (from 100/0 up to 100/30, v/v) as the eluent to provide pure **10** as a black solid, in 47% yield. Mp ˃ 270 ^o^C _(decomp.)_. ^1^H NMR (600 MHz, acetone-*d_6_*) δ 1.58 (s, 3H), 3.66 (d, 2H, *J*=5.7 Hz), 4.35 (t, 1H, D_2_O exch., *J*=5.7 Hz), 5.99 (brs, 1H, D_2_O exch.), 6.67 (d, 1H, *J*=7.4 Hz), 6.71 (d, 1H, *J*=8.4 Hz), 6.98 (brs, 1H, D_2_O exch.), 7.44 (t, 1H, *J*=8.2 Hz), 7.56 (t, 1H, *J*=7.3 Hz), 7.62 (t, 2H, *J*=7.3 Hz), 7.77 (m, 2H), 8.05 (d, 1H, *J*=8.3 Hz), 8.08-8.12 (m, 3H), 8.23 (d, 1H, *J*=8.4 Hz), 8.35 (d, 1H, *J*=8.4 Hz), 9.09 (m, 1H), 9.17 (m, 1H). ^13^C NMR (151 MHz, acetone-*d_6_*) δ 24.66, 67.57, 68.35, 106.27, 106.95, 111.97, 112.02, 112.41, 113.51, 118.83, 123.97, 124.16, 124.94, 127.62, 128.18, 130.27, 130.67, 132.11, 132.74, 133.56, 134.96, 140.41, 142.50, 147.74, 148.05, 151.53, 154.38. HR-MS (ESI) *m/z*: calcd for C_29_H_25_N_6_O, [M1+H]^+^ =473.2084, found 473.2074. Anal. Calcd for C_29_H_24_N_6_O: C, 73.71; H, 5.12; N, 17.78. Found: C, 73.84; H, 5.17; N, 17.61.

**2-(2-Methyl-6-((*E*)-(4-((*E*)-phenyldiazenyl)naphthalen-1-yl)diazenyl)-2,3-dihydro-1*H*-perimidin-2-yl)ethanol (15).**

This compound was prepared according to the general procedure described above, upon reaction of **1** with perimidine **12**. The crude product was purified by column chromatography using a mixture of chloroform / methanol (from 100/1 up to 100/4, v/v) as the eluent to provide pure **15** as a black solid, in 54% yield. Mp 129–131 ^o^C. ^1^H NMR (600 MHz, acetone-*d_6_*) δ 1.60 (s, 3H), 2.13 (m, 2H), 3.83 (m, 1H, D_2_O exch.), 3.88-3.95 (m, 2H), 6.04 (brs, 1H, D_2_O exch.), 6.63 (d, 1H, *J*=7.4 Hz), 6.66 (d, 1H, *J*=8.4 Hz), 7.03 (brs, 1H, D_2_O exch.), 7.44 (t, 1H, *J*=8.2 Hz), 7.58 (m, 1H), 7.65 (t, 2H, *J*=7.5 Hz), 7.76-7.82 (m, 2H), 8.06 (d, 1H, *J*=8.3 Hz), 8.09-8.13 (m, 3H), 8.23 (d, 1H, *J*=8.5 Hz), 8.33 (d, 1H, *J*=8.4 Hz), 9.09 (m, 1H), 9.17 (m, 1H). ^13^C NMR (151 MHz, acetone-*d_6_*) δ 26.91, 43.67, 58.83, 68.03, 106.31, 107.04, 112.01, 112.06, 112.41, 113.54, 119.03, 123.99, 124.17, 124.96, 127.65, 128.22, 130.32, 130.77, 132.15, 132.75, 133.59, 135.04, 140.38, 142.83, 147.74, 148.25, 151.60, 154.42. HR-MS (ESI) *m/z*: calcd for C_30_H_27_N_6_O, [M1+H]^+^ =487.2241, found 487.2232. Anal. Calcd for C_30_H_26_N_6_O: C, 74.05; H, 5.39; N, 17.27. Found: C, 73.96; H, 5.34; N, 17.38.

**3-(2-Methyl-6-((*E*)-(4-((*E*)-phenyldiazenyl)naphthalen-1-yl)diazenyl)-2,3-dihydro-1*H*-perimidin-2-yl)propan-1-ol (16).**

This compound was prepared according to the general procedure described above, upon reaction of **1** with perimidine **13**. The crude product was purified by column chromatography using a mixture of cyclohexane / ethyl acetate (from 1/1 up to 2/8, v/v) as the eluent to provide pure **16** as a black solid, in 50% yield. Mp 124–6 ^o^C. ^1^H NMR (600 MHz, acetone-*d_6_*) δ 1.56 (s, 3H), 1.74-1.80 (m, 2H), 1.92-1.97 (m, 2H), 3.53-3.59 (m, 3H), 6.03 (brs, 1H, D_2_O exch.), 6.63 (d, 1H, *J*=7.3 Hz), 6.66 (d, 1H, *J*=8.5 Hz), 7.06 (brs, 1H, D_2_O exch.), 7.43 (t, 1H, *J*=7.8 Hz), 7.58 (m, 1H), 7.65 (t, 2H, *J*=7.2 Hz), 7.76-7.82 (m, 2H), 8.07 (d, 1H, *J*=8.4 Hz), 8.09-8.13 (m, 3H), 8.23 (d, 1H, *J*=8.4 Hz), 8.31 (d, 1H, *J*=8.4 Hz), 9.09 (m, 1H), 9.17 (m, 1H). ^13^C NMR (151 MHz, acetone-*d_6_*) δ 27.42, 28.02, 38.46, 62.58, 68.02, 106.06, 106.69, 111.77, 112.29, 113.45, 119.09, 123.85, 124.04, 124.85, 127.44, 128.03, 130.11, 130.74, 131.92, 132.59, 133.47, 134.93, 140.19, 142.87, 147.48, 148.34, 151.46, 154.21. HR-MS (ESI) *m/z*: calcd for C_31_H_29_N_6_O, [M1+H]^+^ =501.2397, found 501.2388. Anal. Calcd for C_31_H_28_N_6_O: C, 74.38; H, 5.64; N, 16.79. Found: C, 74.53; H, 5.71; N, 16.61.

**General procedure for the synthesis of esters 11, 17 and 18.**

DCC (93 mg, 0.45 mmol) and DMAP (10 mg, 0.08 mmol) were added under argon, into a suspension of D-biotin (98 mg, 0.40 mmol) in anhydrous dichloromethane (10 mL), and this mixture was stirred at room temperature for 15 minutes, followed by dropwise addition of a dichloromethane solution (6 mL) of the appropriate alcohol **10, 15** or **16** (0.42 mmol). The reaction mixture was stirred at room temperature for 5 days. Then it was diluted with a mixture of CH_2_Cl_2_ / MeOH (10 mL, 100/5, v/v) and filtered through a celite pad. The filtrate was evaporated under reduced pressure and then the crude product was purified by column chromatography, to provide pure esters **11**, **17** or **18**.

**(2-Methyl-6-((*E*)-(4-((*E*)-phenyldiazenyl)naphthalen-1-yl)diazenyl)-2,3-dihydro-1*H*-perimidin-2-yl)methyl 5-((3a*R*,4*R*,6a*S*)-2-oxohexahydro-1*H*-thieno[3,4-*d*]imidazol-4-yl)pentanoate (11, compound** **LG13).**

This compound was prepared according to the general procedure described above, upon reaction of D-biotin with alcohol **10**. The crude product was purified by column chromatography using a mixture of dichloromethane / methanol (from 100/1 up to 100/5, v/v) as the eluent to provide pure **11** as a black solid, in 89% yield. Mp 152–5 ^o^C. ^1^H NMR (600 MHz, DMSO-*d6*) δ 1.14-1.28 (m, 2H), 1.36-1.44 (m, 3H), 1.48-1.56 (m, 4H), 2.08-2.20 (m, 2H), 2.54 (d, 1H, *J*=12.5 Hz), 2.69-2.76 (m, 1H), 2.94-3.01 (m, 1H), 4.00-4.09 (m, 2H), 4.12-4.24 (m, 2H), 6.30 (brs, 1H, D_2_O exch.), 6.36 (brs, 1H, D_2_O exch.), 6.56 (d, 1H, *J*=7.5 Hz), 6.62 (dd, 1H, *J*=8.5 Hz + 2.4 Hz), 6.97 (brs, 1H, D_2_O exch.), 7.42 (t, 1H, *J*=8.1 Hz), 7.61 (t, 1H, *J*=7.2 Hz), 7.67 (t, 2H, *J*=7.3 Hz), 7.80-7.85 (m, 2H), 7.99-8.04 (m, 2H), 8.08 (d, 2H, *J*=7.3 Hz), 8.11 (brs, 1H, D_2_O exch.), 8.15 (d, 1H, *J*=8.3 Hz), 8.18 (dd, 1H, *J*=8.5 Hz + 2.9 Hz), 9.01 (d, 1H, *J*=9.2 Hz), 9.07-9.10 (m, 1H). ^13^C NMR (151 MHz, DMSO-*d6*) δ 24.20, 24.54, 27.97, 28.04, 33.21, 40.05, 55.31, 59.09, 60.94, 65.54, 67.76, 104.97, 105.24, 109.76, 109.96, 111.31, 112.77, 118.90, 122.92, 123.03, 123.78, 127.00, 127.65, 129.58, 130.25, 131.01, 131.51, 131.98, 133.23, 138.41, 141.59, 145.91, 147.46, 150.04, 152.84, 162.63, 172.51. HR-MS (ESI) *m/z*: calcd for C_39_H_39_N_8_O_3_S, [M1+H]^+^ =699.2860, found 699.2849. Anal. Calcd for C_39_H_38_N_8_O_3_S: C, 67.03; H, 5.48; N, 16.03. Found: C, 67.22; H, 5.60; N, 15.94.

**2-(2-Methyl-6-((*E*)-(4-((*E*)-phenyldiazenyl)naphthalen-1-yl)diazenyl)-2,3-dihydro-1*H*-perimidin-2-yl)ethyl 5-((3a*R*,4*R*,6a*S*)-2-oxohexahydro-1*H*-thieno[3,4-*d*]imidazol-4-yl)pentanoate (17).**

This compound was prepared according to the general procedure described above, upon reaction of D-biotin with alcohol **15**. The crude product was purified by column chromatography using a mixture of dichloromethane / methanol (from 100/1 up to 100/5, v/v) as the eluent to provide pure **17** as a black solid, in 58% yield. Mp 139–142 ^o^C. ^1^H NMR (600 MHz, acetone-*d_6_*) δ 1.39-1.47 (m, 2H), 1.55-1.65 (m, 6H), 1.68-1.76 (m, 1H), 2.22-2.26 (m, 2H), 2.28-2.33 (m, 2H), 2.68 (dd, 1H, *J*=12.5 Hz + 4.5 Hz), 2.85-2.90 (m, 1H), 3.12-3.17 (m, 1H), 4.23-4.29 (m, 1H), 4.31-4.35 (m, 2H), 4.42-4.48 (m, 1H), 5.75 (d, 1H, D_2_O exch., *J*=10.2 Hz), 5.79 (d, 1H, D_2_O exch., *J*=7.5 Hz), 6.13 (brs, 0.5H, D_2_O exch.), 6.17 (brs, 0.5H, D_2_O exch.), 6.65 (d, 1H, *J*=7.4 Hz), 6.69 (d, 1H, *J*=8.4 Hz), 7.13 (brs, 0.5H, D_2_O exch.), 7.19 (brs, 0.5H, D_2_O exch.), 7.44 (t, 1H, *J*=7.4 Hz), 7.59 (m, 1H), 7.65 (t, 2H, *J*=7.9 Hz), 7.76-7.82 (m, 2H), 8.06 (d, 1H, *J*=8.3 Hz), 8.09-8.13 (m, 3H), 8.23 (d, 1H, *J*=8.4 Hz), 8.33 (d, 1H, *J*=8.3 Hz), 9.09 (m, 1H), 9.17 (m, 1H). ^13^C NMR (151 MHz, acetone-*d_6_*) δ 25.66, 25.70, 27.80, 29.20, 34.49, 40.08, 41.02, 56.50, 60.94, 61.26, 62.50, 67.41, 106.37, 107.02, 111.97, 112.08, 112.47, 113.57, 188.99, 124.02, 124.21, 124.99, 127.72, 128.27, 130.36, 130.82, 132.22, 132.79, 133.61, 135.01, 140.47, 142.71, 147.83, 148.23, 151.60, 154.45, 163.76, 173.85. HR-MS (ESI) *m/z*: calcd for C_40_H_41_N_8_O_3_S, [M1+H]^+^ =713.3016, found 713.3005. Anal. Calcd for C_40_H_40_N_8_O_3_S: C, 67.39; H, 5.66; N, 15.72. Found: C, 67.56; H, 5.75; N, 15.59.

**3-(2-Methyl-6-((*E*)-(4-((*E*)-phenyldiazenyl)naphthalen-1-yl)diazenyl)-2,3-dihydro-1*H*-perimidin-2-yl)propyl 5-((3a*R*,4*R*,6a*S*)-2-oxohexahydro-1*H*-thieno[3,4-*d*]imidazol-4-yl)pentanoate (18).**

This compound was prepared according to the general procedure described above, upon reaction of D-biotin with alcohol **16**. The crude product was purified by column chromatography using a mixture of dichloromethane / methanol (from 100/1 up to 100/6, v/v) as the eluent to provide pure **18** as a black solid, in 72% yield. Mp 149–151 ^o^C. ^1^H NMR (600 MHz, acetone-*d_6_*) δ 1.35-1.45 (m, 2H), 1.50-1.62 (m, 6H), 1.65-1.74 (m, 1H), 1.85-1.97 (m, 4H), 2.23 (q, 2H, *J*=7.3 Hz), 2.66 (d, 1H, *J*=12.5 Hz), 2.80-2.84 (m, 1H), 3.10-3.15 (m, 1H), 4.02-4.10 (m, 2H), 4.21-4.27 (m, 1H), 4.39-4.44 (m, 1H), 5.74 (brs, 1H, D_2_O exch.), 5.79 (brs, 1H, D_2_O exch.), 6.07 (brs, 0.5H, D_2_O exch.), 6.09 (brs, 0.5H, D_2_O exch.), 6.64 (d, 1H, *J*=7.4 Hz), 6.69 (d, 1H, *J*=8.5 Hz), 7.12 (brs, 0.5H, D_2_O exch.), 7.16 (brs, 0.5H, D_2_O exch.), 7.43 (t, 1H, *J*=7.6 Hz), 7.58 (m, 1H), 7.65 (t, 2H, *J*=7.8 Hz), 7.76-7.82 (m, 2H), 8.06 (d, 1H, *J*=8.4 Hz), 8.09-8.13 (m, 3H), 8.22 (dd, 1H, *J*=8.4 Hz+1.6 Hz), 8.32 (d, 1H, *J*=8.3 Hz), 9.09 (m, 1H), 9.17 (m, 1H). ^13^C NMR (151 MHz, acetone-*d_6_*) δ 24.29, 25.71, 27.39, 29.12, 29.19, 34.32, 37.99, 41.05, 56.56, 60.89, 62.49, 64.76, 68.03, 106.24, 106.85, 111.84, 111.94, 112.43, 113.59, 119.18, 124.01, 124.19, 125.00, 127.69, 128.26, 130.36, 130.88, 132.19, 132.77, 133.63, 135.07, 140.34, 143.00, 147.74, 148.51, 151.66, 154.46, 163.78, 173.75. HR-MS (ESI) *m/z*: calcd for C_41_H_43_N_8_O_3_S, [M1+H]^+^ =727.3173, found 727.3164. Anal. Calcd for C_41_H_42_N_8_O_3_S: C, 67.75; H, 5.82; N, 15.42. Found: C, 67.64; H, 5.78; N, 15.50.

**4-(2,3-dihydro-1*H*-perimidin-2-yl)phenol (14).**

4-Hydroxybenzaldehyde (2.32 g, 18.96 mmol) was added into a solution of 1,8-diaminonaphthalene (**6**, 3 g, 18.96 mmol) in ethanol (15 mL) and this mixture was refluxed for 40 minutes. Upon completion of the reaction, the mixture was allowed to reach room temperature and the precipitate was filtered under vacuum, washed with ethanol (10 mL) and air-dried. Finally, 4.95 g of compound **14** were obtained as an off-white solid. Yield 100%. Mp 169–171 ^o^C (Ref. 172–3 ^o^C) (Popp & Catala, 1964). ^1^H NMR (600 MHz, DMSO-*d6*) δ 5.25 (s, 1H), 6.48 (d, 2H, *J*=7.4 Hz), 6.58 (brs, 2H, D_2_O exch.), 6.81 (d, 2H, *J*=8.5 Hz), 6.97 (d, 2H, *J*=8.1 Hz), 7.14 (t, 2H, *J*=7.7 Hz), 7.41 (d, 2H, *J*=8.5 Hz), 9.48 (brs, 1H, D_2_O exch.). ^13^C NMR (151 MHz, DMSO-*d6*) δ 66.32, 104.22, 112.48, 114.86, 115.13, 126.80, 129.13, 131.96, 134.42, 143.44, 157.73.

**4-(6-((*E*)-(4-((E)-phenyldiazenyl)naphthalen-1-yl)diazenyl)-2,3-dihydro-1*H*-perimidin-2-yl)phenol (19).**

This compound was synthesized following an analogous procedure to that described for the preparation of alcohols **10**, **15** and **16**, upon reaction of (*E*)-4-(phenyldiazenyl)naphthalen-1-amine (**1**) with perimidine **14**. The crude product was purified by column chromatography using a mixture of dichloromethane / ethyl acetate (from 100/0 up to 100/4, v / v) as the eluent to provide pure phenol **19** as a black solid, in 59% yield. Mp ˃ 270 ^o^C _(decomp.)_. ^1^H NMR (600 MHz, DMSO-*d6*) δ 5.59 (s, 1H), 6.68 (d, 1H, *J*=6.9 Hz), 6.72 (d, 1H, *J*=8.5 Hz), 6.87 (d, 2H, *J*=8.5 Hz), 7.07 (brs, 1H, D_2_O exch.), 7.43-7.49 (m, 3H), 7.59 (t, 1H, *J*=7.4 Hz), 7.65 (t, 2H, *J*=7.6 Hz), 7.77-7.84 (m, 2H), 7.99 (d, 1H, *J*=8.4 Hz), 8.04 (d, 1H, *J*=8.4 Hz), 8.08 (d, 2H, *J*=7.5 Hz), 8.17-8.21(m, 2H), 8.24 (d, 1H, *J*=8.4 Hz), 9.01 (m, 1H), 9.10 (m, 1H), 9.61 (brs, 1H, D_2_O exch.). ^13^C NMR (151 MHz, DMSO-*d6*) δ 65.89, 105.23, 105.76, 110.51, 110.81, 111.34, 112.77, 115.09, 118.73, 122.93, 123.04, 123.82, 126.98, 127.60, 129.03, 129.55, 130.07, 131.05, 131.20, 131.46, 132.01, 133.55, 138.64, 143.63, 145.94, 149.10, 150.06, 152.85, 158.05. HR-MS (ESI) *m/z*: calcd for C_33_H_25_N_6_O, [M1+H]^+^ =521.2084, found 521.2078. Anal. Calcd for C_33_H_24_N_6_O: C, 76.14; H, 4.65; N, 16.14. Found: C, 76.02; H, 4.59; N, 16.21.

**4-(6-((*E*)-4-((*E*)-phenyldiazenyl)naphthalen-1-yl)diazenyl)-2,3-dihydro-1*H*-perimidin-2-yl)phenyl 5-((3a*R*,4*R*,6a*S*)-2-oxohexahydro-1*H*-thieno[3,4-*d*]imidazol-4-yl)pentanoate (20).**

This compound was synthesized following an analogous procedure to that described for the preparation of esters **11**, **17** and **18**, upon reaction of phenol **19** with D-biotin. The crude product was purified by column chromatography, using a mixture of dichloromethane / methanol (from 100/0 up to 100/6, v / v) as the eluent to provide **20** as a black solid, in 24% yield. Mp 166–169 ^o^C. ^1^H NMR (600 MHz, acetone-*d6*) δ 1.52-1.60 (m, 2H), 1.64-1.72 (m, 1H), 1.76-1.86 (m, 3H), 2.62 (t, 2H, *J*=7.5 Hz), 2.71 (d, 1H, *J*=12.5 Hz), 2.94 (dd, 1H, *J*=12.5 Hz + 5.1 Hz), 3.22-3.26 (m, 1H), 4.31-4.36 (m, 1H), 4.47-4.51 (m, 1H), 5.71 (s, 1H), 5.75 (brs, 1H, D_2_O exch.), 5.84 (brs, 1H, D_2_O exch.), 6.31 (brs, 1H, D_2_O exch.), 6.77 (d, 1H, *J*=7.4 Hz), 6.81 (d, 1H, *J*=8.4 Hz), 7.18 (brs, 1H, D_2_O exch.), 7.19 (d, 2H, *J*=8.5 Hz), 7.49 (t, 1H, *J*=7.9 Hz), 7.59 (t, 1H, *J*=7.3 Hz), 7.65 (t, 2H, *J*=7.5 Hz), 7.70 (d, 2H, *J*=8.5 Hz), 7.77-7.82 (m, 2H), 8.06 (d, 1H, *J*=8.3 Hz), 8.10-8.13 (m, 3H), 8.25 (d, 1H, *J*=8.4 Hz), 8.42 (d, 1H, *J*=8.4 Hz), 9.09 (m, 1H), 9.18 (m, 1H). ^13^C NMR (151 MHz, acetone-*d6*) δ 25.68, 29.27, 29.31, 34.54, 41.12, 56.52, 60.89, 62.55, 67.75, 106.46, 107.18, 112.59, 112.92, 113.52, 118.38, 122.94, 124.05, 124.24, 124.96, 127.81, 128.30, 129.93, 130.36, 130.51, 132.27, 132.87, 133.57, 135.12, 139.19, 140.93, 144.19, 148.04, 149.28, 149.34, 151.43, 152.64, 154.43, 163.76, 172.61. HR-MS (ESI) *m/z*: calcd for C_43_H_39_N_8_O_3_S, [M1+H]^+^ =747.2860, found 747.2860; calcd for C_43_H_38_N_8_O_3_SNa, [M1+Na]^+^ =769.2680, found 769.2679. Anal. Calcd for C_43_H_38_N_8_O_3_S: C, 69.15; H, 5.13; N, 15.00. Found: C, 69.32; H, 5.22; N, 14.84.

**2,2-Dimethyl-2,3-dihydro-1*H*-perimidine (3).**

A solution of 1,8-diaminonaphthalene (**6**, 4 g, 25.28 mmol) in acetone (14 mL) was stirred at room temperature for 4 days. Upon completion of the reaction, the solvent was evaporated under reduced pressure, diethylether (40 mL) was added into the oily residue and evaporated, causing the crystallization of the perimidine **3**. By this procedure 5 g of the perimidine **16** were obtained, as a beige solid. Yield 100%. Mp 114–116 ^o^C (Ref. 115–116 ^o^C) (Zhang & Zhang, 2007). ^1^H NMR (600 MHz, CDCl_3_) δ 1.46 (s, 6H), 4.14 (brs, 2H, D_2_O exch.), 6.48 (d, 2H, *J*=7.3 Hz), 7.19 (d, 2H, *J*=7.9 Hz), 7.27 (t, 2H, *J*=7.4 Hz + 8.1 Hz). ^13^C NMR (151 MHz, CDCl_3_) δ 28.80, 64.62, 106.06, 113.05, 117.14, 127.12, 134.70, 140.34.

**(*E*)-2-(4-((4-Aminonaphthalen-1-yl)diazenyl)phenyl)ethanol (22).**

2-(4-Aminophenyl)ethanol (**21**, 4 g, 29.16 mmol) was added into a mixture of H_2_O (12 mL) and HCl (10N, 6.4 mL) at 0 ^o^C followed by dropwise addition of an aqueous solution (6 mL) of NaNO_2_ (2.21 g, 32.03 mmol) over a period of 10 minutes and then this mixture was stirred at 0 ^o^C for 100 minutes. Then 0.6 g of sodium acetate trihydrate was added to the solution and stirring was continued for 15 more minutes. This solution of the diazonium salt was added dropwise into a suspension of 1-naphthylamine (4.15 g, 29 mmol) in a mixture of H_2_O (80 mL), EtOH (9 mL) and HCl (10N, 3 mL) over a period of 30 minutes. The deep purple colored suspension was stirred at 0^o^ C for 90 minutes and then at room temperature for 16 hours. The solution was then neutralized with addition of saturated aqueous solution of NaHCO_3_ and the resulting precipitate was filtered under vacuum, washed with H_2_O adequately and air dried. The crude product was purified by column chromatography using a mixture of dichloromethane / ethyl acetate (from 100/5 up to 100/25, v/v) as the eluent to provide 5.2 g of **22** as an orange colored solid. Yield 62%. Mp 110–2^o^ C. ^1^H NMR (600 MHz, DMSO-*d6*) δ 2.81 (t, 2H, *J*=6.9 Hz), 3.67 (q, 2H, *J*=6.9 Hz + 5.5 Hz), 4.69 (t, 1H, D_2_O exch., *J*=5.3 Hz), 6.77 (d, 1H, *J*=8.5 Hz), 6.85 (brs, 2H, D_2_O exch.), 7.38 (d, 2H, *J*=8.1 Hz), 7.48 (t, 1H, *J*=7.8 Hz + 7.3 Hz), 7.62 (t, 1H, *J*=7.9 Hz + 7.3 Hz), 7.80 (d, 2H, *J*=8.1 Hz), 7.88 (d, 1H, *J*=8.5 Hz), 8.20 (d, 1H, *J*=8.5 Hz), 8.90 (d, 1H, *J*=8.5 Hz). ^13^C NMR (151 MHz, DMSO-*d6*) δ 38.82, 62.01, 107.31, 114.78, 121.23, 121.76, 122.61, 122.72, 124.28, 127.29, 129.72, 133.14, 136.84, 141.16, 149.96, 151.60.

**2-(4-((*E*)-(4-((*E*)-(2,2-dimethyl-2,3-dihydro-1*H*-perimidin-6-yl)diazenyl)naphthalen-1-yl)diazenyl)phenyl)ethanol (23).**

This compound was synthesized following an analogous procedure to that described for the preparation of alcohols **10**, **15** and **16**, upon reaction of (*E*)-2-(4-((4-aminonaphthalen-1-yl)diazenyl)phenyl)ethanol (**22**) with perimidine **3**. The crude product was purified by column chromatography using a mixture of dichloromethane / ethyl acetate (from 100/1 up to 100/10, v/v) as the eluent to provide pure alcohol **23** as a black solid, in 67% yield. Mp 249–252 ^o^C _(decomp.)_. ^1^H NMR (600 MHz, DMSO-*d6*) δ 1.48 (s, 6H), 2.86 (t, 2H, *J*=6.9 Hz), 3.71 (q, 2H, *J*=6.9 Hz + 5.1 Hz), 4.74 (t, 1H, D_2_O exch., *J*=5.1 Hz), 6.56 (d, 1H, *J*=6.9 Hz), 6.60 (d, 1H, *J*=8.6 Hz), 6.78 (brs, 1H, D_2_O exch.), 7.43 (t, 1H, *J*=8.1 Hz + 7.7 Hz), 7.48 (d, 2H, *J*=8.4 Hz), 7.76-7.83 (m, 2H), 7.96-8.02 (m, 5H), 8.17 (d, 1H, *J*=8.9 Hz), 8.20 (d, 1H, *J*=8.6 Hz), 9.00 (m, 1H), 9.09 (m, 1H). ^13^C NMR (151 MHz, DMSO-*d6*) δ 28.27, 38.89, 61.82, 64.59, 105.07, 105.52, 109.82, 109.95, 111.21, 112.64, 119.12, 122.78, 123.02, 123.77, 126.83, 127.45, 130.01, 130.27, 130.99, 131.94, 133.51, 138.25, 142.28, 143.83, 145.83, 147.82, 149.95, 151.38. HR-MS (ESI) *m/z*: calcd for C_31_H_29_N_6_O, [M1+H]^+^ =501.2397, found 501.2391. Anal. Calcd for C_31_H_28_N_6_O: C, 74.38; H, 5.64; N, 16.79. Found: C, 74.47; H, 5.68; N, 16.69.

**4-((*E*)-(4-((*E*)-(2,2-Dimethyl-2,3-dihydro-1*H*-perimidin-6-yl)diazenyl)naphthalen-1-yl)diazenyl)phenethyl 5-((3a*R*,4*R*,6a*S*)-2-oxohexahydro-1*H*-thieno[3,4-*d*]imidazol-4-yl)pentanoate (24).**

This compound was synthesized following an analogous procedure to that described for the preparation of esters **11**, **17** and **18**, upon reaction of alcohol **23** with D-biotin. The crude product was purified by column chromatography, using a mixture of dichloromethane / methanol (from 100/0 up to 100/6, v / v) as the eluent to provide **24** as a black solid, in 62% yield. Mp 173–5 ^o^C. ^1^H NMR (600 MHz, acetone-*d6*) δ 1.36-1.43 (m, 2H), 1.55-1.65 (m, 9H), 1.68-1.77 (m, 1H), 2.32 (t, 2H, *J*=7.4 Hz), 2.65 (d, 1H, *J*=12.5 Hz), 2.86 (dd, 1H, *J*=12.5 Hz + 5.1 Hz), 3.09 (t, 2H, *J*=6.7 Hz), 3.12-3.16 (m, 1H), 4.26-4.29 (m, 1H), 4.36-4.42 (m, 3H), 5.66 (brs, 1H, D_2_O exch.), 5.79 (brs, 1H, D_2_O exch.), 6.03 (brs, 1H, D_2_O exch.), 6.62 (d, 1H, *J*=7.4 Hz), 6.65 (d, 1H, *J*=8.5 Hz), 7.04 (brs, 1H, D_2_O exch.), 7.43 (t, 1H, *J*=7.8 Hz), 7.55 (d, 2H, *J*=8.4 Hz), 7.75-7.81 (m, 2H), 8.04-8.08 (m, 3H), 8.10 (d, 1H, *J*=8.4 Hz), 8.22 (d, 1H, *J*=8.4 Hz), 8.33 (d, 1H, *J*=8.4 Hz), 9.09 (m, 1H), 9.17 (m, 1H). ^13^C NMR (151 MHz, acetone-*d6*) δ 25.78, 28.85, 29.29, 34.51, 35.73, 41.09, 56.53, 60.84, 62.49, 64.95, 66.03, 106.21, 106.90, 111.95, 112.11, 112.45, 113.51, 119.03, 124.13, 124.23, 124.97, 127.67, 128.22, 130.77, 130.98, 132.78, 133.57, 135.11, 140.38, 143.10, 143.31, 147.82, 148.46, 151.53, 153.21, 163.70, 173.66. HR-MS (ESI) *m/z*: calcd for C_41_H_42_N_8_O_3_SNa, [M1+Na]^+^ =749.2993, found 749.2992; calcd for C_41_H_43_N_8_O_3_S, [M1+H]^+^ =727.3173, found 727.3172. Anal. Calcd for C_41_H_42_N_8_O_3_S: C, 67.75; H, 5.82; N, 15.42. Found: C, 67.82; H, 5.87; N, 15.34.

**^1^H NMR, ^13^C NMR and HRMS spectra of the target compounds.**

**Compound 10.**

^1^H NMR, acetone-*d_6_*

^13^C NMR, acetone-*d_6_*

HRMS, ESI(+)

**Compound 15.**

^1^H NMR, acetone-*d_6_*

^13^C NMR, acetone-*d_6_*

HRMS, ESI(+)

**Compound 16.**

^1^H NMR, acetone-*d_6_*

^13^C NMR, acetone-*d_6_*

HRMS, ESI(+)

**Compound 19.**

^1^H NMR, DMSO-*d_6_*

^13^C NMR, DMSO-*d_6_*

HRMS, ESI(+)

**Compound 23.**

^1^H NMR, DMSO-*d_6_*

^13^C NMR, DMSO-*d_6_*

HRMS, ESI(+)

**Compound 11.**

^1^H NMR, DMSO-*d_6_*

^13^C NMR, DMSO-*d_6_*

HRMS, ESI(+)

**Compound 17.**

^1^H NMR, acetone-*d_6_*

^13^C NMR, acetone-*d_6_*

HRMS, ESI(+)

**Compound 18.**

^1^H NMR, acetone-*d_6_*

^13^C NMR, acetone-*d_6_*

HRMS, ESI(+)

**Compound 20.**

^1^H NMR, acetone-*d_6_*

^13^C NMR, acetone-*d_6_*

HRMS, ESI(+)

**Compound 24.**

^1^H NMR, acetone-*d_6_*

^13^C NMR, acetone-*d_6_*

HRMS, ESI(+)

HRMS, ESI(+), zoom in

**References**

Crissali P, Kool ET. (2011) Multi-Path Quenchers: Efficient Quenching of Common Fluorophores. Bioconjugate Chem. **22**, 2345-2354.

Popp FD, Catala A J. (1964) Synthesis of potential antineoplastic agents. XI. Some 2-aryl-2,3-dihydro-1H-peri-midines and a perimidine mustard. J Heterocyclic Chem. **1**, 108-109.

Zhang J, Zhang S. (2007) Bismuth(III) Chloride–Promoted Efficient Synthesis of Perimidine Derivatives under Ambient Conditions. Synthetic Commun. **37**, 2615-2624.
