# Supplementary material for: Robust, universal biomarker assay to detect senescent cells in biological specimens
Source: Aging Cell. 2016 Nov 17;16(1):192–7. doi: 10.1111/acel.12545 (PMC5242262; doi:10.1111/acel.12545)
Supplement: Supplementary file 8 — Appendix S2 Biological material, applied staining procedures and notes. [file ACEL-16-192-s008.doc]

**Appendix S2**

**Biological material, applied staining procedures and notes**

**1. Biological material**

A wide range of biological materials that present senescence was utilized to assess the efficacy of the generated compound to reveal such cells, in comparison to the commercially available SBB reagent. This material consisted of *in vitro* and *in viv*o models and clinical samples, known to exhibit robust cellular senescence, either by means of proliferative exhaustion (Replicative Senescence, RS) or upon stress induction (Stress Induced Senescence, SIPS), including oncogenic stress signals (Oncogene Induced Senescence, OIS) (Georgakopoulou *et al*., 2013; Liakou *et al*., 2016; Galanos *et al*., 2016; Petrakis *et al*., 2016; Bartkova *et al*., 2006; Liontos *et al*., 2007; Liontos *et al*., 2009; Hellevik & Martinez-Zubiaurre, 2014). The employed senescence models (summarized in **Supplementary Fig. 3**) were the following:

**1.1 *In vitro* models**

The *in vitro* models comprised seven cellular systems (**Supplementary Fig. 3**). The first one was primary human diploid lung fibroblasts (DLFs) at late passages, exhibiting replicative senescence and were compared to early proliferative passages devoid of senescence (Georgakopoulou *et al*., 2013; Liakou *et al*., 2016). The same cellular system was used at early passages that underwent SIPS upon γ-irradiation and was compared in relation to non irradiated counterparts. Another set of models comprised two inducible Saos2 Tet-ON osteosarcoma cell lines carrying p53 and p21^WAF1/Cip1^, respectively, two well established effectors of senescence (Georgakopoulou *et al*., 2013; Galanos *et al*., 2016). Finally, the U2OS-E2F1 ER, U2OS-Cdt1 Tet-ON and HBECs-Cdc6 Tet-ON inducible systems that undergo SIPS due to oncogenic stress signals were also utilized (Petrakis *et al*., 2016; Liontos *et al*., 2007; Liontos *et al*., 2009). Parental cell lines were obtained either directly from ATCC or from collaborating laboratories that generated them. In addition, the human cancer cell line HeLa and near normal Li-Fraumeni fibroblasts (Galanos *et al*., 2016) were also employed as control cell lines for the specificity of staining of the SBB compounds.

**1.2 *In vivo* models**

The *in vivo* material included tissues from three mouse models and three human clinical settings.

***1.2.1 Animal models***

In the first mouse model, the K-ras^V12^ oncogene is conditionally activated in the lung, generating adenomas (preneoplastic lesions) and adenocarcinomas, as previously reported (Collado *et al*., 2005). Senescence occurs in adenoma cells, as an anti-tumor barrier (Bartkova *et al*., 2006), while in adenocarcinomas it is bypassed and therefore not detected (Georgakopoulou *et al*., 2013; Collado *et al*., 2005).

Secondly, a mouse model of bleomycin induced pneumopathy was generated, as previously described (Aoshiba *et al*., 2013). In brief, C57BL/6 mice were intratracheally injected with 30 ul of PBS or with 30 ul of a solution containing 2.5 mg/kg (mouse weight) of bleomycin. Two weeks later mice were sacrificed and lungs were collected, fixed and embedded in paraffin blocks. Bleomycin administration is well known to induce at initial stages robust senescence in alveolar epithelial cells, while prolonged treatment (over two weeks) results in gradual interstitial lung fibrosis as a consequence of a SASP (Senescence Associated Secretory Phenotype) phenomenon (Aoshiba *et al*., 2013).

The third mouse model is a xenograft one, in which tumors were generated by the subcutaneous injection of 1E-6 Mel9 human melanoma cells in athymic nude mice. After tumor development, mice were injected intravenously (everyday, for 2 weeks) with 200 ul vehicle or with 200 ul of a solution containing 50 mg/kg palbociclib (Pfizer). Palbociclib acts as a Cdk4/6 inhibitor that has been recently demonstrated to inhibit melanoma progression through senescence induction (Yoshida *et al*., 2016).

***1.2.2 Human clinical samples***

The clinical settings used in the current study included tissue samples from patients suffering from head/neck (Evangelou *et al*., 2013) and breast carcinomas that were irradiated to achieve shrinkage of the tumors prior to surgery. Irradiation is well established to induce senescence either *in vitro* (in cells isolated from solid tumors) or *in vivo* affecting different cellular compartments of solid tumors, including cancer associated fibroblasts (CAFs) (Liakou *et al*., 2016; Hellevik & Martinez-Zubiaurre, 2014; Gewirtz, 2014). In the case of irradiated breast tumors we recently demonstrated that ionizing radiation provokes premature senescence of stromal fibroblasts *in vivo* (Liakou *et al*., 2016). Corresponding non-irradiated cases served as negative controls. The third model employed, consisted of a set of congenital nevi. These melanocytic lesions are well known to exhibit robust senescence as an antitumor barrier, induced via activation of the DNA damage response (Halazonetis *et al*., 2008; Michaloglou *et al*., 2005). We also included in the analysis a limited number of cases of melanomas that developed either on pre-existing or in the vicinity of congenital nevi. This is a very useful tool since, as previously described, when senescence is bypassed in melanoma cells (Michaloglou *et al*., 2005), no evidence of senescent markers expression is obtained. In this setting, we were able to evaluate and directly compare the staining results in a positive for senescence preoneoplastic lesion (congenital nevi) and in neoplastic lesion (melanoma), negative for senescence, as both conditions were located in the same tissue section. Generally, all findings were always compared to the corresponding adjacent normal epidermis relative to the location of melanocytes.

A set of reference tissues were also employed for the initial screening to reveal among the new generated chemical compounds those with the most optimal performance for senescence detection. These tissues are known to exhibit high cellular content of lipofuscin that makes them ideal *in vivo* positive controls and were obtained from:

i*. Liver tissue from young and aged patients.* Liver tissue from five (three male and two female) and seven (four male and three female) specimens from young and old individuals respectively, were analyzed. Age of young patients ranged from 18-23 years, while the corresponding one for old patients was 66-80 years. The material consisted of two surgical segments and three biopsies for the young cases while the corresponding one for the old cases included three surgical segments and four biopsies. For the young individuals the material was obtained in the frame of non pathological conditions (liver transplantation donors and surgical manipulations to deal with abdominal injury). Liver tissues from old individuals came from the surgical margins of hepatectomy segments and biopsies that were performed during clinical investigations.

*ii. Seminal vesicles included in prostatectomy segments with prostatic cancer.*

Tissues from patients with benign prostatic hyperplasia (BPH), a condition related to senescence were also employed (Castro *et al*., 2013). Adjacent normal prostatic glands served as negative controls.

Protocols for animal tissues and clinical sample collection, and their experimental use were approved by the Bio-Ethics Committee of Medical School of Athens, in accordance with the Declaration of Helsinki and local laws and regulations, following also written consent from the patients in the case of clinical samples.

**2. Staining Method**

**2.1 Preparation of the biological material**

***2.1.1 Materials:***

1. Cells (From Aspiration or Cell Culture)

2. Cover Slips

3. Various fixative media such as 100% Methanol, 100% Ethanol, and 1-5% (w/v) Parafolmadehyde/PBS Solution can be applied

4. 1-5% (w/v) Parafolmadehyde/PBS Solution: Dissolve 1-5 gr of Parafolmadehyde (PFH) in 100ml of PBS in a glass beaker. Heat and stir the mixture until it becomes transparent (**Notes 1-3**). Let the solution to cool and adjust pH to 7.4 (**Notes 1-3**)

5. Phosphate Buffered Saline (PBS; 10×): 1.37 M NaCl, 27 mM KCl, 100 mM

Na_2_HPO_4_, 18 mM KH_2_PO_4_, pH 7.4

6. Incubation Chambers for Cover Slips

7. Tissue samples (Fixed in 10% Buffered Formalin Solution and Paraffin Embedded, FFPE)

8. 10% Buffered Formalin Solution (Sigma-Aldrich)

9. 1% (v/v) formaldehyde/PBS: Add in a volumetric cylinder, of appropriate size, 10 ml of Buffered Formalin Solution 10%, adjust volume to 100 ml with PBS and stir at room temperature (RT). Store at 4°C

10. Positively Charged Glass Slides

11. Coplin Jars

12. Glass Beaker

13. Volumetric Cylinder

14. Thin edged Forceps

***2.1.2 Procedures:***

1. Cells. Mount cells on cover slips and fix them in 1-5% (w/v) parafolmadehyde/PBS solution for 5 min at RT. Then wash three times (approx.1 min) with PBS (**Notes 1-2**).

2. Tissue samples (FFPE sections): Cut thin paraffin sections and mount them on positively charged glass slides. Store at 40 ºC until staining.

**2.2. Preparation of the compound (GL13) solution**

***2.2.1 Substances:***

1. Compound (GL13)

2. 100% ethanol (100% EtOH)

3. Parafilm

4. Fritted glass filter of medium porosity

5. Airtight Dye Container

***2.2.2 Procedures:***

1. Dissolve 40 mg of the compound (GL13) in 7.4 ml 100% EtOH in a glass beaker and cover it with parafilm.
2. Incubate at 56^o^C in a waterbath for 120 min until the compound is completely dissolved.

**2.3. Compound (GL13) staining method**

***2.3.1 Materials:***

1. Xylene

2. Gradually Decreased (96%, 80%, 70%, 50%) EtOH Solutions

3. Syringe

4. 13mm filter, membrane 0.22μm

5. Soft paper (dry or dipped in ethanol)

6. Anti-biotin antibody ([Hyb-8] ab201341, Abcam)

7. Ultravision Quanto Detection System HRP DAB kit (Cat no: TL-125-QHD)

8. Glycerol (Sigma-Aldrich)

9. Tris Buffered Saline (TBS; 10×): 1.5 M NaCl, 0.1 M Tris–HCl, pH 7.4. Store at 4°C.

10. 0.5% Triton X/TBS: 0.5ml Triton X diluted in 99.5ml TBS

11. Mounting media Glycergel (DakoCytomation).

12. Light Microscope

13. Anti-p21^WAF1/Cip1^ mouse primary antibody [(F-5)(sc-6246) Santa-Cruz]

14. Anti-p16^INK4A^ mouse primary antibody [(sc-74401) Santa-Cruz]

15. Anti-Ki67 rabbit polyclonal antibody [ ab16667, Abcam]

16. Secondary goat anti-mouse antibody Alexa Fluor 568 conjugated [Thermo Fisher Scientific (Cat no: A-11004)]

17. DAPI (4',6-Diamidino-2-Phenylindole, Dihydrochloride) [Thermo Fisher Scientific (Cat no: D1306)]

18. Fluorescence microscope

***2.3.2 Procedures:***

2.3.2.1. Staining protocol for cells mounted on coverslips

1. Wash x1 in TBS for 5 min at RT.
2. Block the endogenous hydrogen peroxidase, using the UltraVision Hydrogen Peroxide Block included in the Ultravision Quanto Detection System HRP DAB kit (Cat no: TL-125-QHD) for 10 min at RT and in dark conditions (**Note 5**).
3. Wash x2 in TBS for 30 sec and x1 for 5 min at RT.
4. Wash x1 in EtOH 50% for 5 min at RT.
5. Wash x1 EtOH 70% for 5 min at RT.
6. Incubate with the compound (GL13) at RT. A drop of freshly prepared compound (GL13) is placed on a clean slide with the use of a syringe that carried a 45μm filter (*see also Figure 1 from Evangelou & Gorgoulis, 2016*). Then the cover-slip with the cells is turned down (using thin edged forceps) on the slide and attached on the dye drop in a way that the material faces down the drop on the slide (**Notes 6,7**), (*see also Figure 1 from Evangelou & Gorgoulis, 2016*).

7. The staining reaction is monitored under the light microscope until detection of the signal (average time 5-8 min) **(Notes 8-11**).

8. Wash x2 in EtOH 50% **(Note 12**).

9. Repeat washing x2 in fresh EtOH 50% **(Note 12**).

10. Wash x2 in TBS for 30 sec and x1 for 5min at RT.

11. Incubate with 0.5% Triton X/TBS for 3min at RT.

12. Wash x1 in TBS for 5min at RT.

13. Incubate with the primary anti-biotin antibody diluted 1:500 in TBS ([Hyb-8] ab201341, Abcam) for 60min at 37^o^C (**Note 6 and 13**).

14. Wash x3 in TBS for 5min at RT.

15. Incubate with the Primary antibody amplifier Quanto included in the Ultravision Quanto Detection System HRP DAB kit (Cat no: TL-125-QHD), for 10min at RT (**Note 6**).

16. Wash x3 in TBS for 5min at RT.

1. Incubate with the HRP Polymer Quanto included in the Ultravision Quanto Detection System HRP DAB kit (Cat no: TL-125-QHD), for 10min at RT.
2. Wash x2 in TBS for 30 sec and x1 for 5min at RT (**Note 6**).
3. Application of DAB Plus Chromogen diluted 1/300 in DAB Plus Substrate [Ultravision Quanto Detection System HRP DAB kit (Cat no: TL-125-QHD)]. The staining reaction is monitored under the light microscope until detection of the dark brown signal (average time 30 sec-1 min) at RT 1.
4. Wash in tap water for 5 min at RT.
5. Counterstain with Hematoxylin diluted 1:4 in deionized water
6. Wash in tap water for 5 min at RT.
7. Apply permanent mounting media Glycergel (DakoCytomation).
8. Observation under the light microscope (**Notes 9-11**).

2.3.2.2. Staining protocol for tissue sections mounted on glass slides

1. Deparaffinize sections by:
   1. Incubation at 60^o^C for 30min.
   2. Washing in Xylene for 15min at RT.
2. Gradual rehydration in:
   1. EtOH 100% for 15min at RT.
   2. EtOH 96% for 10min at RT.
   3. EtOH 80% for 5min at RT.
   4. EtOH 70% for 3min at RT.
   5. EtOH 50% for 3min at RT.
3. Wash x1 in TBS for 5 min at RT.
4. Block endogenous hydrogen peroxidase, using the UltraVision Hydrogen Peroxide Block included in the Ultravision Quanto Detection System HRP DAB kit (Cat no: TL-125-QHD) for 10 min at RT and in dark conditions (**Note 6**).
5. Wash x2 in TBS for 30 sec and x1 for 5 min at RT (**Note 14**).
6. Wash x1 in EtOH 50% for 5 min at RT.
7. Wash x1 EtOH 70% for 5 min at RT.
8. Incubate with the compound (GL13) at RT. A drop of freshly prepared compound (GL13) is placed on a clean slide with the use of a syringe that carrying a 2mm filter (*see also Figure 1 from Evangelou & Gorgoulis, 2016*). Then the cover-slip with the cells is turned down (using thin edged forceps) on the slide and attached on the dye drop in a way that the material faces down the drop on the slide (**Notes: 6, 7**), (*see also Figure 1 from Evangelou & Gorgoulis, 2016*).
9. The staining reaction is monitored under the light microscope until detection of the signal (average time 5-8 min), (**Notes 8-11**).
10. Wash x2 in EtOH 50%. (**Note: 12**).
11. Repeat x2 in fresh EtOH 50% (**Note: 12**).
12. Wash x2 in TBS for 30 sec and x1 for for 5min at RT.
13. Incubate with 0.5% Triton X/TBS for 3min at RT.
14. Wash x1 in TBS for 5min at RT.
15. Incubate with the primary anti-biotin antibody diluted 1:500 in TBS ([Hyb-8] ab201341, Abcam) for 60min at 37^o^C (**Note 6, 13**).
16. Wash x3 in TBS for 5min at RT.
17. Incubate with the Primary antibody amplifier Quanto included in the Ultravision Quanto Detection System HRP DAB kit (Cat no: TL-125-QHD), for 10min at RT (**Note 6**).
18. Wash x3 in TBS for 5min at RT.
19. Incubate with the HRP Polymer Quanto included in the Ultravision Quanto Detection System HRP DAB kit (Cat no: TL-125-QHD), for 10min at RT (**Note 6**).
20. Wash x2 in TBS for 30 sec and x1 for 5min at RT.
21. Apply DAB Plus Chromogen diluted 1/300 in DAB Plus Substrate [Ultravision Quanto Detection System HRP DAB kit (Cat no: TL-125-QHD)]. The staining reaction was monitored under the light microscope until detection of the dark brown signal (average time 30 sec-1 min) at RT.
22. Wash in tap water for 5 min at RT.
23. Counterstain with Hematoxylin diluted 1:4 in deionized water.
24. Wash in tap water for 5 min at RT.
25. Apply permanent mounting media Glycergel (DakoCytomation).
26. Observation under the light microscope (**Notes 9-11**).

2.3.2.3. Co-staining protocol for cells mounted on coverslips

1. Wash x1 in TBS for 5 min at RT.
2. Block the endogenous hydrogen peroxidase, using the UltraVision Hydrogen Peroxide Block included in the Ultravision Quanto Detection System HRP DAB kit (Cat no: TL-125-QHD) for 10 min at RT and in dark conditions (**Note 6**).
3. Wash x2 in TBS for 30 sec and x1 for 5 min at RT.
4. Incubate with Ultra Protein Block included in the Ultravision Quanto Detection System HRP DAB kit (Cat no: TL-125-QHD) for 7 min in RT.
5. Wash x1 in TBS for 5 min at RT.
6. Wash with 0.5% Triton X/TBS for 3 min at RT.
7. Wash x1 in TBS for 5 min at RT.
8. Incubate with primary antibody (**Note 18**).
9. Wash x2 in TBS for 30 sec and x1 for min RT.
10. Incubate with the Primary antibody amplifier Quanto included in the Ultravision Quanto Detection System HRP DAB kit (Cat no: TL-125-QHD), for 10min at RT.
11. Wash x3 in TBS for 5 min at RT.
12. Incubate with the HRP Polymer Quanto included in the Ultravision Quanto Detection System HRP DAB kit (Cat no: TL-125-QHD), for 10min at RT (**Note 6**).
13. Wash x2 in TBS for 30 sec and x1 for 5 min at RT.
14. Apply DAB Plus Chromogen diluted 1/100 in DAB Plus Substrate [Ultravision Quanto Detection System HRP DAB kit (Cat no: TL-125-QHD)]. The staining reaction was monitored under the light microscope until detection of the dark brown signal (average time 30 sec-1 min) at RT.
15. Wash in tap water for 5 min at RT.
16. Wash x1 in EtOH 50% for 5 min at RT.
17. Wash x1 EtOH 70% for 5 min at RT.
18. Incubate with the compound (GL13) at RT. A drop of freshly prepared compound (GL13) is placed on a clean slide with the use of a syringe that carried a 2mm filter (*see also Figure 1 from Evangelou & Gorgoulis, 2016*). Then the cover-slip with the cells is turned down (using thin edged forceps) on the slide and attached on the dye drop in a way that the material faces down the drop on the slide (**Notes: 6, 7**), (*see also Figure 1 from Evangelou & Gorgoulis, 2016*).
19. The staining reaction is monitored under the light microscope until detection of the signal (average time 5-8 min) **(Notes 8-11**).
20. Wash x3 in EtOH 50% **(Note 12**).
21. Repeat washing x3 in fresh EtOH 50% **(Note 12**).
22. Wash x2 in TBS for 30 sec and x1 for 5min at RT.
23. Incubate with the anti-biotin antibody diluted 1:500 in TBS ([Hyb-8] ab201341, Abcam) for 60min at 37^o^C (**Note 6 and 13**).
24. Wash x3 in TBS for 5min at RT.
25. Incubate with the secondary goat anti-mouse antibody AP conjugated (Invitrogen, Cat no: G21060) diluted 1:800 in TBS, for 60 min at RT (**Note 6**).
26. Wash x3 in TBS for 5min at RT.
27. Wash x2 in TBS for 30 sec and x1 for 5min at RT (**Note 6**).
28. Application of NBT/BCIP substrate [dilute 1 tablet in 10 ml ddH_2_O (Roche kit, Cat no: REF 11 697 471 001)] with the addition of 20 μl of 100 mM Levamisol. The staining reaction is monitored under the light microscope until detection of the dark blue signal (average time 5-7min) at RT.
29. Wash x2in KTBT buffer for 5 min at RT
30. Wash in tap water for 5 min at RT.
31. Wash in tap water for 5 min at RT.
32. Apply permanent mounting media Glycergel (DakoCytomation).
33. Observation under the light microscope (**Notes 9-11, 19**).

2.3.2.4. Co-staining protocol for tissue sections mounted on glass slides

1. Deparaffinize sections by:
   1. Incubation at 60^o^C for 30min.
   2. Washing in Xylene for 15min at RT.
2. Gradual rehydration in:
   1. EtOH 100% for 15min at RT.
   2. EtOH 96% for 10min at RT.
   3. EtOH 80% for 5min at RT.
   4. EtOH 70% for 3min at RT.
   5. EtOH 50% for 3min at RT.
3. Wash x1 in TBS for 5 min at RT.
4. Block endogenous hydrogen peroxidase, using the UltraVision Hydrogen Peroxide Block included in the Ultravision Quanto Detection System HRP DAB kit (Cat no: TL-125-QHD) for 10 min at RT and in dark conditions (**Note 6**).
5. Wash x2 in TBS for 30 sec and x1 for min at RT.
6. Blocking with Ultra V Block for 7 min at RT.
7. Wash x1 in TBS for 5 min at RT.
8. Wash with 0.5% Triton X/TBS for 3 min at RT.
9. Wash x1 in TBS for 5 min at RT.
10. Incubate with primary antibody (**Note 18**).
11. Wash x2 in TBS for 30 sec and x1 for min RT.
12. Incubate with the Primary antibody amplifier Quanto included in the Ultravision Quanto Detection System HRP DAB kit (Cat no: TL-125-QHD), for 10min at RT.
13. Wash x3 in TBS for 5 min at RT.
14. Incubate with the HRP Polymer Quanto included in the Ultravision Quanto Detection System HRP DAB kit (Cat no: TL-125-QHD), for 10min at RT (**Note 6**).
15. Wash x2 in TBS for 30 sec and x1 for 5 min at RT.
16. Apply DAB Plus Chromogen diluted 1/300 in DAB Plus Substrate [Ultravision Quanto Detection System HRP DAB kit (Cat no: TL-125-QHD)]. The staining reaction was monitored under the light microscope until detection of the dark brown signal (average time 30 sec-1 min) at RT.
17. Wash in tap water for 5 min at RT.
18. Wash x1 in EtOH 50% for 5 min at RT.
19. Wash x1 EtOH 70% for 5 min at RT.
20. Incubate with the compound (GL13) at RT. A drop of freshly prepared compound (GL13) is placed on a clean slide with the use of a syringe attached to a 2mm filter (*see also Figure 1 from Evangelou & Gorgoulis, 2016*). Subsequently, the cover-slip with the cells is turned down (using thin edged forceps) on the slide and attached on the dye drop in a way that the material faces down the drop on the slide (**Notes 6, 7**), (*see also Figure 1 from Evangelou & Gorgoulis, 2016*).
21. The staining reaction is monitored under the light microscope until detection of the signal (average time 5-8 min) **(Notes 8-11**).
22. Wash x3 in EtOH 50% **(Note 12**).
23. Repeat washing x3 in fresh EtOH 50% **(Note 12**).
24. Wash x2 in TBS for 30 sec and x1 for 5min at RT.
25. Incubate with the anti-biotin antibody diluted 1:500 in TBS ([Hyb-8] ab201341, Abcam) for 60min at 37^o^C (**Note 6 and 13**).
26. Wash x3 in TBS for 5min at RT.
27. Incubate with the secondary goat anti-mouse antibody AP conjugated (Invitrogen, Cat no: G21060) diluted 1:800 in TBS, for 60 min at RT (**Note 6**).
28. Wash x3 in TBS for 5min at RT.
29. Wash x2 in TBS for 30 sec and x1 for 5min at RT (**Note 6**).
30. Application of NBT/BCIP substrate [dilute 1 tablet in 10 ml ddH_2_O (Roche kit, Cat no: REF 11 697 471 001)] with the addition of 20 μl of 100 mM Levamisol. The staining reaction is monitored under the light microscope until detection of the dark brown signal (average time 5-7min) at RT.
31. Wash x2in KTBT buffer for 5 min at RT
32. Wash in tap water for 5 min at RT.
33. Wash in tap water for 5 min at RT.
34. Apply permanent mounting media Glycergel (DakoCytomation).
35. Observation under the light microscope (**Notes 9-11, 19**).

2.3.2.5. Immunofluorescence staining protocol for cells mounted on coverslips

1. Wash x1 in TBS for 5 min at RT.

2. Wash x1 in EtOH 50% for 5 min at RT.

3. Wash x1 EtOH 70% for 5 min at RT.

4. Incubate with the compound (GL13) at RT. A drop of freshly prepared compound (GL13) is placed on a clean slide with the use of a syringe that carrying a 2mm filter (*see also Figure 1 from* *Evangelou & Gorgoulis, 2016*). Then the cover-slip with the cells is turned down (using thin edged forceps) on the slide and attached on the dye drop in a way that the material faces down the drop on the slide (**Note: 6, 7**), (*see also Figure 1 from Evangelou & Gorgoulis, 2016*).

5. The staining reaction is monitored under the light microscope until detection of the signal (average time 5-8 min) **(Notes 8-11**).

6. Wash x2 in EtOH 50% **(Note 12**).

7. Repeat washing x2 in fresh EtOH 50% **(Note 12**).

8. Wash x2 in TBS for 30 sec and x1 for 5min at RT.

9. Incubate with 0.5% Triton X/TBS for 3min at RT.

10. Wash x1 in TBS for 5min at RT.

11. Incubate with the primary anti-biotin antibody diluted 1:400 in TBS ([Hyb-8] ab201341, Abcam) for 60min at 37^o^C (**Note 6 and 13**).

12. Wash x3 in TBS for 5min at RT.

13. Incubate with goat anti-mouse secondary antibody, 568 Alexa Fluor conjugated, diluted 1:200 in TBS [Thermo Fisher Scientific (Cat no: A-11004)] for 60 min in RT.

14. Wash x5 in TBS for 5min at RT.

15. Incubate with DAPI diluted 1:1000 in TBS for 5min at RT.

16. Wash x2 in TBS for 5min at RT.

17. Apply permanent mounting medium Glycergel (DakoCytomation).

18. Observe under the fluorescence microscope (**Notes 9-11, 15**).

**2.4 Technical Notes on section 2:**

1. Follow accurately all safety regulations (wear gloves, mask and glasses) during manipulations and waste disposal instructions when disposing waste materials.

2. All solutions must be prepared using deionized water (unless otherwise indicated).

3. Preparation of the Parafolmadehyde/PBS Solution must be performed in a fume hood to avoid any contact with fumes. Preferably always prepare a fresh solution before the experiments.

4. During the entire process the dye container must be air tightly sealed to prevent evaporation of ethanol, which in turn leads to precipitation of the saturated dye solution in cells and tissues.

5. Store the GL13 dye solution in a non-light absorbing and airtight container at room temperature for a short period of time (maximum 2-3 months). Upon longer intervals between experiments preferentially prepare a fresh solution of the dye.

6. All incubations were performed in chambers (cover slips) and coplin jars (glass slides) to avoid exsiccation of the material.

7. This step is crucial to avoid evaporation of the dye.

8. Absence of staining with the compound (GL13) “per se” within 5-8 minutes does not always indicate that the sample is negative for senescence. From our experience we suggest to proceed with the DAB visualization reaction. In many cases, especially in FFPE tissues, despite it was challenging to detect the positive granules after **GL13** histochemical staining, we clearly detected positive (dark brown) senescent cells after completion of the DAB reaction. The addition of the chromogenic assay increases dramatically the sensitivity of the method, possibly due to lowering of the detection threshold. A possible reason for above is that the smaller granules in FFPE tissue might occur due to partial lipid striping of lipofuscin during sample preparation.

9. The observation that intraluminar, intracellular or extracellular mucous droplets can occasionally stain light blue when the compound is used, should always be taken into consideration.

10. Cautious should be taken when analyzing material enriched in macrophages. These cells are often positive either upon histochemical staining with the compound “per se” or when the complete method is performed and can be mistakenly considered as senescent cells. A similar phenomenon has been described by others for alveolar macrophages that showed positive staining when SA-β-galactosidase activity, the most widely used senescent marker in fresh material (Dimri *et al*., 1995), was applied (Aoshiba *et al*., 2003). Attention should also be paid not to misinterpret melanin deposits within melanocytic lesions with the compound stained granules, as the first exhibit mostly larger diameters.

11. Omission of the compound (GL13) should always be performed as a negative control experiment.

12. This step is crucial to remove and estimate "background dirt" and clean cover slips and slides.

13. Omission of the primary anti-biotin antibody should always serve as negative control.

14. In the case of liver tissue the following steps were additionally performed after step 5 (Section 2.3.2.2):

- Incubation with blocking biotin (streptavidin included in the Streptavidin/Biotin blocking kit, SP 2002, Vector) for 15min at RT

- Washing in TBS x2 for 30 sec and x1 for 5min at RT

- Incubation with blocking biotin (biotin included in the Streptavidin/Biotin blocking kit, SP 2002, Vector) for 15min at RT

- Washing x2 in TBS for 30 sec and x1 for 5min at RT

15. The Fluorescent Microscope can be used in control experiments. Lipofuscin that accumulates in senescent cells is well known to exhibit autofluorescent properties that are quenched not only with SBB (Georgakopoulou *et al*., 2013), but also by the current compound (GL13) staining. We used a Leica DMRAZ microscope equipped with a Leica DFC350FX camera. The sample was mounted in 40% glycerol/TBS medium, after its appropriate preparation and observed by excitation at 450-490 nm, using a dichromatic mirror at 510 nm and a long-pass filter at 515 nm (Georgakopoulou *et al*., 2013).

16. The method is also applicable on frozen OCT embedded material. If preservation of morphology is not crucial, the slides can be directly stained as described above for cells (section 2.3.2.1). If preservation of morphology is essential, material can be fixed in 1% (w/v) formaldehyde/PBS for 5 min and after x3 washings (approx. for1 min each) in PBS, and stained by continuing the procedure from step 3 in section 2.3.2.2 and on.

17. In case of using GL13 *per se* in cells and tissues use 0.1% Nuclear Fast Red as counterstain. Preparation of the dye solution has been previously described (Georgakopoulou *et al*., 2013; Liakou *et al*., 2016; Galanos *et al*., 2016; Evangelou & Gorgoulis, 2016).

18. In double staining experiments anti-p21^WAF1/CIP1^, p16^INK4A^ and Ki67 antibodies were diluted: 1:200, 1:100 and 1:500 in TBS, respectively, and incubated overnight at 4^o^C.

19. Counterstain in double staining reaction is omitted to avoid interference with the chromogen signals.

**3. Sudan Black-B staining method**

The Sudan Black-B staining method for cells and tissues has been previously described in detail by our group (Georgakopoulou *et al*., 2013; Liakou *et al*., 2016; Galanos *et al*., 2016; Evangelou & Gorgoulis, 2016).

**4. Flow cytometry analysis for detection of senescent cells in cell culture**

1. Cells harvested from culture are fixed in EtOH 70% at 4^o^C.

2. Centrifuge at 1200 rpm for 5 min at RT.

3. Wash x1 in 5ml PBS at RT.

4. Centrifuge at 1200 rpm for 5 min at RT.

5. Incubate in 0.1% Triton X/PBS for 15 min at RT.

6. Centrifuge at 1200rpm for 5 min at RT.

7. Wash x1 EtOH 50% for 5 min at RT

8. Centrifuge at 1200 rpm for 5 min at RT.

9. Wash x1 EtOH 70% for 5 min at RT.

10. Centrifuge at 1200 rpm for 5 min at RT.

11. Incubate with compound GL13 for 8 min at RT.

12. Centrifuge at 1200 rpm for 5 min at RT.

13. Wash with EtOH 50% and centrifuge at 1200 rpm for 5 min at RT, repeat x3.

14. Wash x1 in PBS.

15. Centrifuge at 1200 rpm for 5 min at RT.

16. Incubate with the primary anti-biotin antibody diluted 1:400 in PBS ([Hyb-8] ab201341, Abcam) for 60min at 37^o^C

17. Wash x1 with PBS.

18. Centrifuge at 1200 rpm for 5 min at RT.

19. Incubate with secondary antibody, goat anti-mouse 568 Alexa Fluor conjugated, diluted 1:100 in PBS [Thermo Fisher Scientific (Cat no: A-11004)] for 20-30 min in dark and on ice.

20. Wash x1 with PBS.

21. Centrifuge at 1200 rpm for 5 min at RT.

**Supplementary References**

Aoshiba K, Tsuji T, Kameyama S, Itoh M, Semba S, Yamaguchi K, Nakamura H. (2013) Senescence-associated secretory phenotype in a mouse model of bleomycin-induced lung injury. Exp Toxicol Pathol. **65**, 1053-62.

Aoshiba K, Tsuji T, Nagai A. (2003) Bleomycin induces cellular senescence in alveolar epithelial cells. Eur Respir J. **22**, 436-43

Castro P, Giri D, Lamb D, Ittmann M. (2003) Cellular senescence in the pathogenesis of benign prostatic hyperplasia. Prostate **55**, 30–38.

Collado M, Gil J, Efeyan A, Guerra C, Schuhmacher AJ, Barradas M, Benguría A, Zaballos A, Flores JM, Barbacid M, Beach D, Serrano M. (2005) Tumour biology: senescence in premalignant tumours. Nature **436**, 642.

Evangelou K, Bartkova J, Kotsinas A, Pateras IS, Liontos M, Velimezi G, Kosar M, Liloglou T, Trougakos IP, Dyrskjot L, Andersen CL, Papaioannou M, Drosos Y, Papafotiou G, Hodny Z, Sosa-Pineda B, Wu XR, Klinakis A, Ørntoft T, Lukas J, Bartek J, Gorgoulis VG. (2013) The DNA damage checkpoint precedes activation of ARF in response to escalating oncogenic stress during tumorigenesis. Cell Death Differ. **20**, 1485-97.

Evangelou K, Gorgoulis VG. (2016) Methods in Molecular Biology, vol. 1534, Springer.

Gewirtz DA. (2014) Autophagy and senescence in cancer therapy. J Cell Physiol. **229**, 6-9.

Hellevik T, Martinez-Zubiaurre I. (2014) Radiotherapy and the Tumor Stroma: The Importance of Dose and Fractionation. Front Oncol. **4**, 1.

Michaloglou C, Vredeveld LC, Soengas MS, Denoyelle C, Kuilman T, van der Horst CM, Majoor DM, Shay JW, Mooi WJ, Peeper DS. (2005) BRAFE600-associated senescence-like cell cycle arrest of human naevi. Nature **436**, 720–724.

Yoshida A, Lee EK, Diehl JA. (2016) Induction of Therapeutic Senescence in Vemurafenib-Resistant Melanoma by Extended Inhibition of CDK4/6. Cancer Res. **76**, 2990-3002.

Debacq-Chainiaux F1, Erusalimsky JD, Campisi J, Toussaint O. (2009) Protocols to detect senescence-associated beta-galactosidase (SA-betagal) activity, a biomarker of senescent cells in culture and in vivo. Nat Protoc. **4**, 1798-806.
